# Anchoring Vertical Dipole to Enable Efficient Charge Extraction for High‐Performance Perovskite Solar Cells

**DOI:** 10.1002/advs.202203640

**Published:** 2022-09-04

**Authors:** Heng Liu, Zhengyu Lu, Weihai Zhang, Jiantao Wang, Zhengli Lu, Quan Dai, Xingnan Qi, Yueqing Shi, Yuhui Hua, Rui Chen, Tingting Shi, Haiping Xia, Hsing‐Lin Wang

**Affiliations:** ^1^ School of Materials Science and Engineering Harbin Institute of Technology Harbin 150001 P. R. China; ^2^ Department of Materials Science and Engineering Southern University of Science and Technology Shenzhen Guangdong 518055 P. R. China; ^3^ Shenzhen Grubbs Institute and Department of Chemistry Southern University of Science and Technology Shenzhen Guangdong 518055 P. R. China; ^4^ Guangdong Provincial Engineering Technology Research Center of Vacuum Coating Technologies and New Energy Materials Department of Physics Jinan University Guangzhou Guangdong 510632 P. R. China; ^5^ Department of Electrical and Electronic Engineering Southern University of Science and Technology Shenzhen Guangdong 518055 P. R. China; ^6^ Key University Laboratory of Highly Efficient Utilization of Solar Energy and Sustainable Development of Guangdong Southern University of Science and Technology Shenzhen Guangdong 518055 P. R. China

**Keywords:** built‐in electric field, dipole moment, organic–inorganic (OI) complexes, perovskite solar cells, two‐step sequential method

## Abstract

Perovskite solar cells (PSCs) via two‐step sequential method have received great attention in recent years due to their high reproducibility and low processing costs. However, the relatively high trap‐state density and poor charge carrier extraction efficiency pose challenges. Herein, highly efficient and stable PSCs via a two‐step sequential method are fabricated using organic—inorganic (OI) complexes as multifunctional interlayers. In addition to reduce the under‐coordinated Pb^2+^ ions related trap states by forming interactions with the functional groups, the complexes interlayer tends to form dipole moment which can enhance the built‐in electric field, thus facilitating charge carrier extraction. Consequently, with rational molecular design, the resulting devices with a vertical dipole moment that parallels with the built‐in electric field yield a champion efficiency of 23.55% with negligible hysteresis. More importantly, the hydrophobicity of the (OI) complexes contributes to an excellent ambient stability of the resulting device with 91% of initial efficiency maintained after 3000 h storage.

## Graphical abstract

Highly efficient and stable perovskite solar cells (PSCs) via the two‐step sequential method are fabricated using organic–inorganic (OI) complexes as multifunctional interlayers. OIcomplexes not only passivate the metal ions related trap states but also introduce dipole moment which can enhance the built‐in electric field, thus facilitating charge carrier extraction. The resulting devices perform a champion efficiency of 23.55% with excellent long‐term air stability.

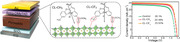

## 1 Introduction

Organic–inorganic halide perovskite solar cells (PSCs) have attracted tremendous attention in recent years due to its excellent photovoltaic properties.^[^ ^1^, ^2^, ^3^, ^4^ ^]^ Evolution of device efficiency from 3.8% to 25.5% has been witnessed within the past 13 years.^[^ ^5^ ^]^ Currently, most PSCs with certified efficiency over 24% are exclusively based on the antisolvent‐assisted one‐step solution method, which is environmentally unfriendly.^[^ ^6^, ^7^, ^8^, ^9^ ^]^ In contrast, two‐step sequential method exhibits high reproducibility, low solvent incompatibility, and good flexibility, which are essential for practical applications.^[^ ^10^, ^11^, ^12^, ^13^, ^14^ ^]^ However, the corresponding perovskite films suffer from high trap‐state density, especially at the carrier transport layer (CTL)/perovskite interfaces, leading to relatively low device efficiency. Therefore, effective strategies that optimize the corresponding interfaces are urgently needed.

Surface passivation is a widely adopted strategy to modify the interfaces between the CTL and perovskite.^[^ ^15^, ^16^, ^17^, ^18^ ^]^ It has been reported that organic materials with electron‐rich carbonyl or amino functional groups can effectively passivate the under‐coordinated metal ions, contributing to improved device efficiency.^[^ ^19^, ^20^, ^21^, ^22^, ^23^ ^]^ For example, Qiu et al. adopted carbonyl‐rich cinnamic acid as passivator to functionalize perovskite surface for efficient and stable PSCs. They indicated that the carbonyl groups can strongly interacted with under‐coordinated Pb^2+^ ions, delivering perovskite films with low trap states.^[^ ^24^ ^]^ Similarly, in our previous work, 1,8‐octanediamine dihydroiodide (ODADI) was adopted to develop an alkylammonium predeposition strategy for the fabrication of high‐quality perovskite film. It is found that the predeposited ODADI layer not only facilitates the diffusion of organic salts via interaction with PbI_2_, but also passivates the buried‐interface defects, resulting in perovskite film with low defect density, high crystallinity, and superior electronic properties.^[^ ^25^ ^]^ Despite the significant reduction of defect density after surface passivation, the limited charge extraction efficiency at the interfaces hinders further improvement of device performance.

Recently, novel materials with strong electric dipole moments were adopted to facilitate charge extraction, thus improving device performance.^[^ ^26^, ^27^, ^28^ ^]^ For instance, Zhang et al. introduced organic donor‐*π*‐acceptor (D‐*π*‐A) molecules with dipole moments at the perovskite/electron transport layer (ETL) interface. The novel molecules can enhance the built‐in electric field, which drives the electrons to ETL and repels the holes to the hole transport layer (HTL), delivering an enhancement of device efficiency from 18.8% to 21.4%.^[^ ^29^ ^]^ Further, Canil et al. managed to tune the work function (WF) of halide perovskite materials by using self‐assembled monolayers of small molecules which induce stable dipole moments at the perovskite surface.^[^ ^30^ ^]^ These studies have demonstrated that the internal dipole can effectively optimize the interfacial energy band structure, enhance the built‐in electric field, and improve charge extraction, contributing to high device performance. However, a comprehensive study that disclose the effect of dipole moment direction on device performance remains lacking. Besides, it is expected that a well‐designed interlayer material which not only provides dipole moment but also passivates nonradiative recombination centers can further enhance device performance.

Herein, two types of novel organic–inorganic (OI) complexes (CL‐CH_3_ and CL‐CF_3_) were rationally designed and introduced as interlayer between perovskite and HTL. The results suggest that both CL‐CH_3_ and CL‐CF_3_ can simultaneously reduce under‐coordinated Pb^2+^ ions related trap states by forming coordination through carbonyl groups, and facilitate the charge extraction with the introduction of dipole moment. While, due to the coexistence of the anchoring effect derived from trifluoromethyl (‐CF_3_) and carbonyl (‐C=O) groups, CL‐CF_3_ complex layer form a vertical dipole moment that aligned with the built‐in electric field of the PSCs, leading to much stronger photoexcited holes extraction ability. As a result, the fabricated devices with CL‐CF_3_ interlayer yield a champion PCE of 23.55%. More importantly, the hydrophobicity of CL‐CF_3_ complex contributes to an excellent ambient stability of the resulting device with 91% of initial efficiency maintained after 3000 h storage. This novel OI complex interlayer provides a new direction toward more versatile passivators for highly efficient and stable PSCs.

## 2 Results and Discussion

**Figure 1:**
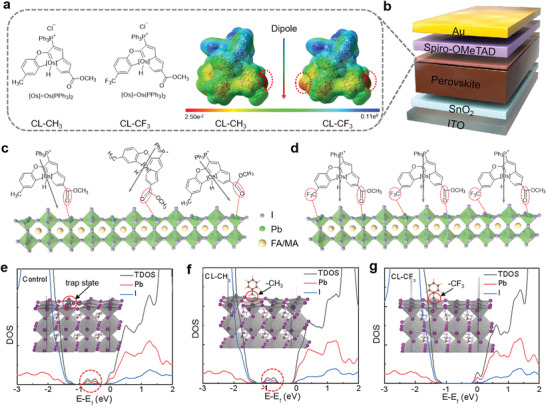
a) Chemical structures and corresponding simulated electrostatic potential profiles of CL‐CH_3_ and CL‐CF_3_. b) Schematic of the device structure. c,d) Schematic illustration of molecular orientation of CL‐CH_3_ and CL‐CF_3_ on perovskite.) The density of states of (e) control perovskite film surface with anti‐site Pb_I_ defects and passivated by (f) CL‐CH_3_ and (g) CL‐CF_3_.

To achieve multifunctional interlayer, two types of OI complexes (CL‐CH_3_, CL‐CF_3_) were rationally designed and synthesized. Corresponding synthetic procedures were detailly described in the Experimental Section. The nuclear magnetic resonance (NMR), Mass spectroscopy (MS), and single‐crystal X‐ray diffraction (XRD) characterizations of CL‐CH_3_ and CL‐CF_3_ are shown in Figures S1–S10, Supporting Information. Thermogravimetric analyses (TGA) of CL‐CH_3_ and CL‐CF_3_ show that the initial decomposition temperatures (T5) as measured at the point of 5% weight loss are 217.1 °C and 207.6 °C (Figure S11, Supporting Information), respectively. The chemical structures and simulated electrostatic potential profiles of the complexes were presented in **Figure** **1**a, in which it is found that both CL‐CH_3_ and CL‐CF_3_ show obvious molecular dipoles. According to density functional theory (DFT), CL‐CF_3_ exhibits a significantly higher dipole moment of 11.04 D than that of CL‐CH_3_ (8.4 D) due to the strong electron‐withdrawing Trifluoromethyl (–CF_3_) group, as shown in Figure S12 (Supporting Information). The favorable thermal and electronic properties of OI complexes make it an ideal candidate to serve as an interlayer with a device structure of indium tin oxide (ITO)/ tin oxide (SnO_2_)/ perovskite/ OI complexes/ Spiro‐OMeTAD/ Au, as shown in Figure 1b.

It has been reported that the electron‐withdrawing group tends to coordinate with under‐coordinated Pb^2+^ ions in perovskite films, thus providing a driving force for molecular assembly.^[^ ^31^ ^]^ In this case, the CL‐CH_3_ and CL‐CF_3_ complexes form a dipole interlayer with its negative end pointed towards perovskite, and positive end pointed outward. As shown in Figure 1a, for CL‐CH_3_ molecule, the electron lacking area (positive blue area) is mainly located around Os atoms, and the electron‐rich area (negative red area) is located around –C=O group. Compared with CL‐CH_3_, CL‐CF_3_ has a very different pattern of charge distribution as the electron‐rich areas are located around –C=O and –CF_3_ groups. In contrast to CL‐CF_3_, CL‐CH_3_ molecule with a electron‐rich –C=O group such that the dipole moment points away from the perovskite surface, as shown in Figure 1c. While, CL‐CF_3_ molecule with two electron‐withdrawing groups (–C=O and –CF_3_) can interact with perovskite vertically as shown in Figure 1d. Further, The density of states of perovskite film surface structures and two models passivated by CL‐CH_3_ and CL‐CF_3_ molecules were calculated respectively and depicted correspondingly in Figure 1e–g. It is found that anti‐site Pb_I_ defects of the perovskite film surface is obviously trap state shown in Figure 1e. After passivated by CL‐CH_3_ and CL‐CF_3_ molecules, a clean gap appeared again in the passivation structure after doping CL‐CF_3_ molecules (Figure 1g), but little change in trap state after doping CL‐CH_3_ molecules can be observed (Figure 1f).

A series of spectroscopy probes were used to verify the interaction (passivation effect) between OI complex and perovskite films. Figure S13 (Supporting Information) shows the Fourier transform infrared spectroscopy (FTIR) spectra for the pure CL‐CH_3_, CL‐CF_3_, PbI_2_, PbI_2_+CL‐CH_3_, and PbI_2_+CL‐CF_3_ that dissolved in DMSO. It is noted that the stretching vibration of C=O bond shifted from 1660 cm^−1^ in pure OI complexes (CL‐CH_3_, CL‐CF_3_) to a lower wavenumber of 1643 cm^−1^ for the PbI_2_+CL‐CH_3_ and PbI_2_+CL‐CF_3_ samples, suggesting an interaction between PbI_2_ and –C=O group in OI complexes.^[^ ^32^, ^33^, ^34^ ^]^ Further, X‐ray photoelectron spectroscopy (XPS) was adopted to analyze the chemical compositions and environments of the perovskite films. The appearance of Os, P, and F characteristic peaks shown in Figure S14 (Supporting Information) indicates the existence of OI complexes in the final film. In addition, the binding energy shift of the Pb 4f and F1s shown in Figure S15 (Supporting Information) further indicates the interaction between OI complexes and the perovskite film.^[^ ^35^ ^]^ Both theoretical and experimental results suggest that CL‐CH_3_ complex tends to interact with perovskite film through lone pairs on –C=O functional group, while the interaction between CL‐CF_3_ complex and perovskite film derives from –C=O and ‐CF_3_ functional groups.

To study the effect of OI complexes interlayer on the crystallinity of the perovskite films, X‐ray diffraction (XRD) measurement was conducted. The corresponding XRD patterns exhibit similar diffraction peaks located around, 14, 24, and 28°, which are assigned to the (110), (202), and (220) planes of the FAPbI_3_ phase, respectively, while the peak at 12.6° belongs to the (001) plane of PbI_2_ (Figure S16, Supporting Information). In order to determine whether FAI, MABr, and MACl penetrated to the bottom of PbI_2_, we conducted Helios Nanolab 600i FIB and FEI Talos transmission electron microscope (TEM) with Super‐X EDS, as shown in Figure S17, Supporting Information. In this study, the organic salts we adopted were FAI, MABr, and MACl, all of which contain N atoms. It can be clearly seen that the N element is distributed throughout the perovskite layer, indicating that the FAI, MABr, and MACl penetrated to the bottom of PbI_2_. Further, Scanning electron microscopy (SEM), atomic force microscope (AFM), UV‐vis absorption spectrum, and corresponding Tauc's plot characterizations were conducted, as shown in Figures S18–S21 (Supporting Information). The results suggest that the OI complexes will not affect the morphology and optical bandgap of perovskite films, maintaining its excellent optoelectronic properties.

**Figure 2:**
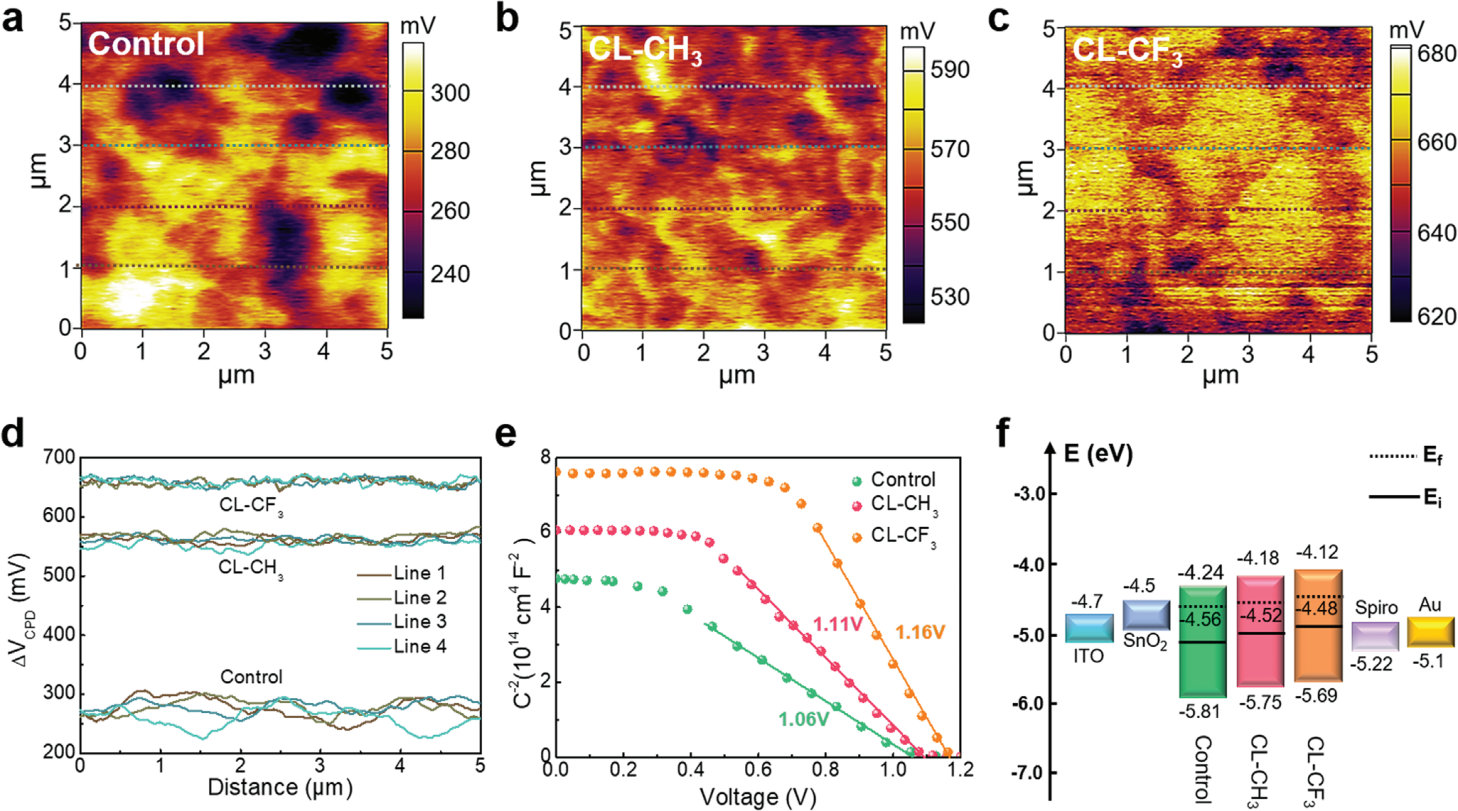
a‐c) KPFM of control, CL‐CH_3,_ and CL‐CF_3_ films. d) Contact potential difference (ΔV_CPD_) of control, CL‐CH_3,_ and CL‐CF_3_ films. e) Mott–Schottky plots of PSCs based on control, CL‐CH_3,_ and CL‐CF_3_ films. f) Energy‐level diagram constructed from UPS results, where *E*_f_ is fermi level, and *E*_i_ is intrinsic Fermi level.

Kelvin probe force microscopy (KPFM) was performed to study the local surface potential of the perovskite films with and without CL‐CH_3_, CL‐CF_3_ treatment (denoted as control, CL‐CH_3_ and CL‐CF_3_ film hereafter, respectively), as shown in **Figure** **2**a–c. According to the line profile results shown in Figure 2d, the mean contact potential difference (CPD) of the films increased from 270 mV for control to 560 mV for CL‐CH_3_ and 660 mV for CL‐CF_3_, respectively. It is known that the work functions (WFs) of sample can be estimated from the tip work function by subtracting the measured CPD value. In this case, the largest CPD value derived from CL‐CF_3_ film delivers to a smallest work function due to the introduction of vertical dipole moment.^[^ ^36^ ^]^ Besides, it can be clearly observed that the OI complexes treated perovskite films reveal smaller CPD variation within 20 mV than that of the control film (100 mV) across the sample, inferring a mitigation of potential difference between grains and grain boundaries, which can be ascribed to the reduced defects at the surface due to coordination effect.^[^ ^37^ ^]^ Figure 2e presents the Mott–Schottky plots of the devices based on different perovskite films. The built‐in electric field (*V*_bi_) can be derived from the intercept of the *X*‐axis according to the equation$\;\;\frac{1}{C^{2}}=\frac{2(V_{bi}-V)}{\varepsilon \varepsilon_{0}qA^{2}N}\;$, where *C* is the measured capacitance, *ε* is the relative permittivity, *ε*_0_ is the vacuum permittivity, *A* is the active area of the device, *N* is the doping density of the donor, and *V* is the applied potential.^[^ ^38^ ^]^ Accordingly, the calculated *V*_bi_ of control, CL‐CH_3,_ and CL‐CF_3_ films are 1.05, 1.11, and 1.16 V, respectively. The enhancement of *V*_bi_ after OI complexes treatment is mainly attributed to the introduction of dipole moment. While the largest *V*_bi_ derived from CL‐CF_3_ film further verifying the synchronized dipole moment and built‐in electric field, thus facilitating charge carrier extraction.^[^ ^39^, ^40^ ^]^

The electronic structures of the perovskite films were investigated using ultraviolet photoelectron spectroscopy (UPS). Figure S22 (Supporting Information) shows the obtained secondary electron cutoff (*E*_cutoff_) and onset (*E*_onset_) energy of the control, CL‐CH_3,_ and CL‐CF_3_ films. Combining with the optical bandgap derived from UV‐vis absorption (Figure S21, Supporting Information), the conduction band minimum (CBM) and valence band maximum (VBM) of the films were ascertained, and the corresponding parameters are summarized in Table S1 (Supporting Information). It is noted that the perovskite films reveal a decreased WF from 4.56 to 4.52 then to 4.48 eV for the control, CL‐CH_3,_ and CL‐CF_3_ film, respectively, which is consistent with the KPFM results. The reduction of WFs for OI complexes treated films is mainly attributed to the introduction of dipole moment, which benefits to charge extraction. Besides, as depicted in Figure 2f, based on the relative position between Fermi level (*E*_f_) and intrinsic Fermi level (*E*_i_), the control film reveals *n*‐type self‐doped, which might be attributed to the halide vacancies that act as electron donors. Interestingly, after the introduction of OI complexes interlayer, a dedoping process occurred, leading to more intrinsic perovskite film which has led to less charge recombination and thus higher device efficiency.^[^ ^41^ ^]^ Additionally, it is worth noting that CL‐CF_3_ film shows a smallest energy difference of 0.09 eV between perovskite/ETL (0.38 eV) and perovskite/HTL (0.47 eV) when compared with CL‐CH_3_ (0.21 eV) film and control film (0.33 eV). This favorable energy difference contributes to a well‐balanced charge extraction, which is essential for enhancing device performance and mitigating hysteresis.^[^ ^42^ ^]^

**Figure 3:**
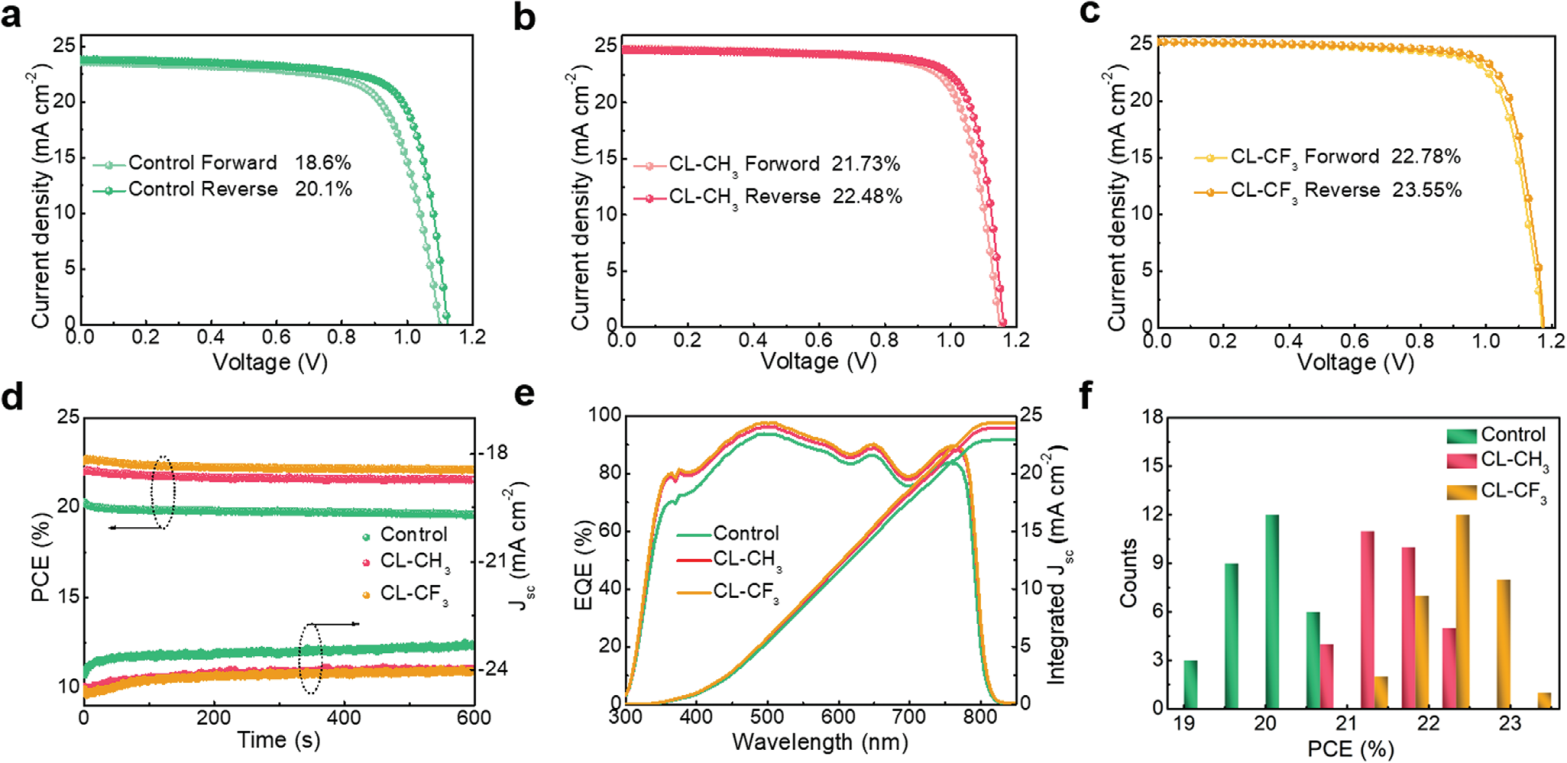
a‐c) *J–V* curves from reverse (*V*_oc_ to *J*_sc_) and forward (*J*_sc_ to *V*_oc_) scan of the devices derived from control, CL‐CH_3,_ and CL‐CF_3_ films, respectively. d) Steady‐state power outputs of the devices. e) EQE and integrated *J*_sc_ spectra of the devices. f) Statistical PCE parameters of 30 independent devices based on control, CL‐CH_3,_ and CL‐CF_3_ films.

To verify the effectiveness of OI complexes interlayer on device performance, PSCs based on control, CL‐CH_3,_ and CL‐CF_3_ films were fabricated. **Figure** **3**a–c shows the current density–voltage (*J–V*) curves of the champion PSCs based on control, CL‐CH_3,_ and CL‐CF_3_ perovskite film, respectively, and the corresponding photovoltaic parameters are summarized in **Table** **1**. The devices based on OI complexes treated perovskite films reveal significant improvement on open‐circuit voltage (*V*_oc_) and short‐circuit current density (*J*_sc_), contributing to an enhancement on power conversion efficiency (PCE) from 20.1% to over 22%. The much improved *V*_oc_ is mainly ascribed to the reduction of under‐coordinated Pb^2+^ ions related to nonradiative recombination. While, higher *J*_sc_ can be attributed to the enhancement of charge carrier extraction due to the introduction of dipole moment. Besides, it should be noted that CL‐CF_3_ film‐based device has a higher PCE of 23.55% than that of the CL‐CH_3_ film‐based device (22.48%), which is mainly attributed to the anchored vertical dipole moment induced by CL‐CF_3_ complex, as discussed earlier. In addition, hysteresis studies based on H‐index (HI): HI = (PCE_reverse_ − PCE_forward_)/PCE_reverse_, where PCE_reverse_ and PCE_forward_ are efficiencies of devices for reverse and forward scan, respectively. The above results suggest that OI complexes treated devices deliver a smaller HI (less than 4%) than that of control (7.5%), which is mainly attributed to the well‐balanced charge extraction due to the modified energetics.

**Table 1:** Photovoltaic performance of the champion devices for the corresponding PSCs

Device	Scan direction	*V*_oc_ [V]	*J*_sc_ [mA cm^–2^]	FF [%]	PCE [%]	Integrated *J*_sc_ [mA cm^–2^]	HI [%]
	Forward	1.09	23.60	71.8	18.6		
Control	Reverse	1.12	23.80	75.3	20.1	22.93	7.5
	Forward	1.15	24.84	76.8	21.73		
CL‐CH_3_	Reverse	1.16	24.72	78.3	22.48	23.97	3.3
	Forward	1.17	25.22	77.2	22.78		
CL‐CF_3_	Reverse	1.18	25.24	79.2	23.55	24.42	3.2

To confirm the reliability of the *J–V* measurements, steady‐state power output (SPO) at the maximum power was recorded as shown in Figure 3d. The PCE of the devices based on control, CL‐CH_3_ and CL‐CF_3_ film stabilized at 19.85%, 21.80%, and 22.45%, respectively, which are consistent with the values obtained from *J–V* measurements. Figure 3e shows the external quantum efficiency (EQE) spectra of the devices, from which the integrated *J*_sc_ located at 22.93, 23.97, and 24.42 mA cm^−2^ for the control, CL‐CH_3,_ and CL‐CF_3_ devices, respectively, agreeing well with the measured value. The statistical histogram of the PCEs derived from 30 independent PSCs were presented in Figure 3f, from which it can be observed that devices based on different perovskite films reveal high reproducibility with a narrow PCE distribution. Besides, CL‐CF_3_ devices produce a highest average PCE of 22.5%, which is in accordance with the *J–V* measurements.

**Figure 4:**
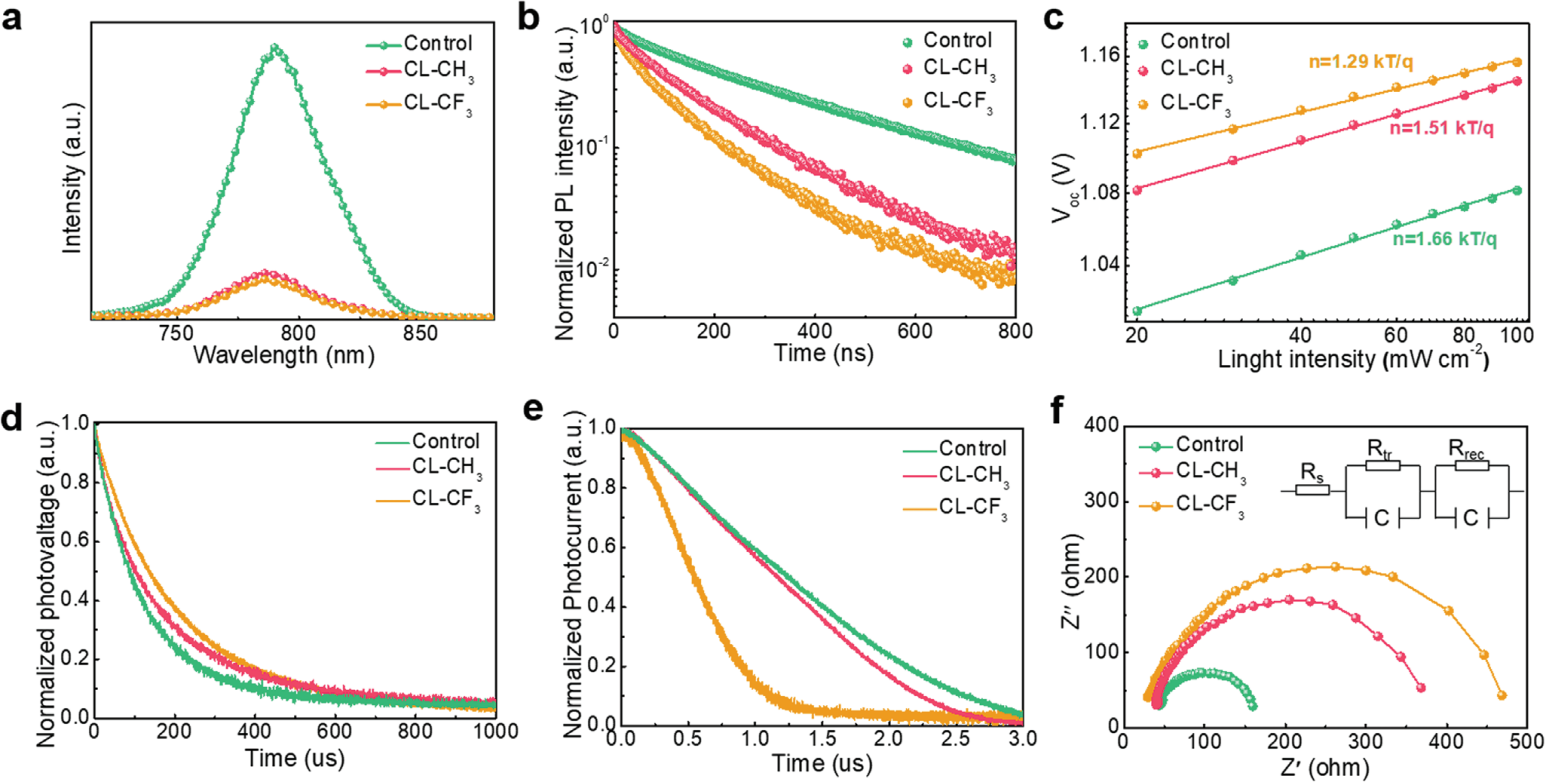
a) Steady‐state photoluminescence (PL) spectra of the perovskite films with the charge transport layer. b) Time‐resolved photoluminescence (TRPL) spectra of corresponding perovskite films. c) Incident light intensity dependence of *V*_oc_ for devices based on different films. d) TPV and e) TPC decay curves of the devices. f) Nyquist plots for the different devices.

To better understand the mechanisms behind the excellent device performance, the charge dynamics of the devices were systematically studied. **Figure** **4**a shows the steady‐state photoluminescence (PL) spectra of different perovskite films with a charge transfer layer. It is clear that after OI complexes treatment, the perovskite film performs much quicker PL quenching than control film, indicates a more efficient charge carrier extraction. Considering the fact that all films possess a same ETL (SnO_2_), the enhancement of charge extraction is mainly attributed to a more effective photoexicted hole extraction due to the introduction of dipole moment and modified energy level between perovksite/HTL interface. The corresponding time‐resolved PL (TRPL) spectra were presented in Figure 4b, and the results were fitted by a biexponential decay function with detailed parameters summarized in Table S2 (Supporting Information). The shorter carrier lifetime of CL‐CF_3_ film (113.5 ns) than CL‐CH_3_ film (153.1 ns), which means a more effective charge extraction due to the stronger built‐in electronic field (Figure 2e related discussion) that induced by the superimposed dipole moment.^[^ ^43^, ^44^ ^]^

Figure 4c presents the relationship between *V*_oc_ and light intensity of the devices, in which the deviation of the calculated slope from unity kT q^–1^ indicates the trap‐assisted recombination in PSCs.^[^ ^45^ ^]^ In this case, the reduction of slope value from 1.66 kT q^–1^ for control device to 1.51 kT q^–1^ for CL‐CH_3_ device and 1.29 kT q^–1^ for CL‐CF_3_ device suggests that the trap‐assisted recombination within the OI complexes treated perovskite film was substantially suppressed, which can be ascribed to the passivation effect. More importantly, the smaller slope derived from CL‐CF_3_ device (1.29 kT q^–1^) indicates a stronger passivation effect resulting from the coexistence of the anchoring effect from –CF_3_ and –C=O groups in CL‐CF_3_ film. Further, the space‐charge‐limited current (SCLC) technique was adopted to evaluate the defect density of different perovskite films. Figure S23a (Supporting Information) illustrates the typical dark *J–V* curves of electron‐only devices with the structure of ITO/SnO_2_/Perovskite/PCBM/Ag. It can be seen that the *V*_TFL_ of control, CL‐CH_3_, and CL‐CF_3_ film‐based devices are 0.86, 0.65, and 0.54 V, respectively. According to $N_{t}=\frac{2V_{TFL}\varepsilon_{r}\varepsilon_{0}}{eL^{2}}\;$, where *ε_r_* is the relative dielectric constant of perovskite, which is 62.23,^[^ ^46^ ^]^ *ε_0_* is the vacuum permittivity, e is the elementary charge, and *L* is the thickness of the perovskite film, which is ≈500 nm according to Figure S17 (Supporting Information), it can be calculated that the trap‐state density of control, CL‐CH_3_, and CL‐CF_3_ films are 2.37×10^16^, 1.79×10^16^, and 1.48×10^16^ cm^–3^, respectively. Besides, hole‐only devices with the structure of ITO/NiOx/Perovskite/ Spir‐OMeTAD/Au were also fabricated and investigated (Figure S23b, Supporting Information). The hole mobility of the films was further estimated using the Mott‐Gurney equations at the SCLC region. After careful calculation, the hole mobility of control, CL‐CH_3_, and CL‐CF_3_ films are 3.8 × 10^–2^, 4.2 × 10^–2^, and 5.7 × 10^–2^ cm^–2^V^–1^S^–1^, respectively. The results suggest that CL‐CF_3_ complex can effectively improve the hole mobility of the film. The lowest trap‐state density of CL‐CF_3_ film agrees well with the smallest trap‐assisted recombination.^[^ ^47^ ^]^

Figure 4d shows the transient photovoltage (TPV) curves of the devices, from which the photovoltage decay time of the control (154.3 µs), CL‐CH_3_ (205.7 µs), and CL‐CF_3_ (224.4 µs) devices were obtained. A longer photovoltage decay time indicates a slower charge carrier recombination process. Accordingly, OI complexes treatment contributes to suppressed charge carrier recombination, which is responsible for the enhancement of *V*_oc_.^[^ ^48^ ^]^ Meanwhile, transient photocurrent (TPC) measurement was conducted to evaluate charge transportation and extraction efficiency in different devices (Figure 4e). Apparently, CL‐CF_3_ film‐based device performs a fastest current decay of 0.497 µs, which is almost five times faster than that of control device (2.26 µs), indicating much improved carrier extraction efficiency.^[^ ^49^ ^]^ Furthermore, electrochemical impedance spectroscopy (EIS) measurement was adopted to study the interfacial charge transfer and recombination of the devices. Corresponding Nyquist plots were measured under a bias of 1 V under dark condition at room temperature. Figure 4f shows the Nyquist plots of the devices, and the inset presents the corresponding equivalent circuit model, which include series resistance (*R*_s_), chemical capacitance (*C*), transfer resistance (*R*_tr_), and recombination resistance (*R*_rec_). In general, the high‐frequency component can be ascribed to the R_tr_ and the low‐frequency component is attributed to the *R*_rec_. In the high‐frequency region, the *R*_tr_ for the control, CL‐CH_3_, and CL‐CF_3_ devices are 48, 39, and 28 Ω, respectively. The smallest *R*_tr_ derived from CL‐CF_3_ device can be ascribed to the significantly enhanced charge carrier extraction efficiency due to the regulated vertical dipole moment direction. In the low‐frequency region, the *R*_rec_ for the control, CL‐CH_3_, and CL‐CF_3_ devices can be fitted as 156, 368, and 469 Ω, respectively. The largest *R*_rec_ value for the CL‐CF_3_ device indicates that the charge recombination process within the device is effectively inhibited.^[^ ^50^ ^]^ Overall, the enhanced charge carrier extraction, reduced trap‐state density, and suppressed charge carrier recombination are the main reasons for the enhancement of photovoltaic performance in the OI complexes treated devices.

**Figure 5:**
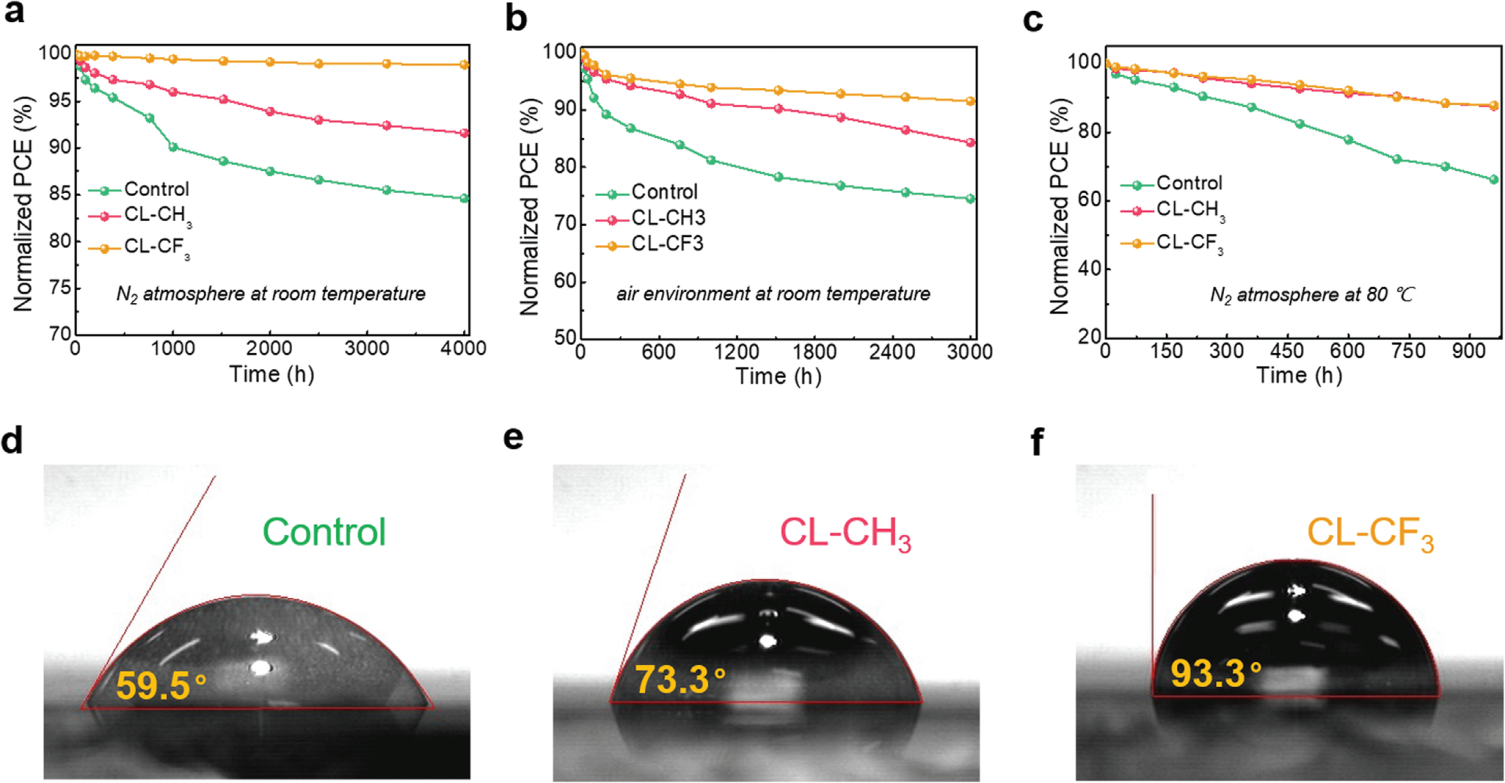
Stability measurements of unencapsulated devices based on different films in a) glovebox at room temperature, b) ambient environment with RH of 20%–30%, c) glovebox at 85 °C. d‐f) Water contact angle results of different perovskite films.

In addition to device efficiency, stability is another important indicator for evaluating device performance. Figure S24, Supporting Information, presents the operational stability of the unencapsulated devices under continuous 1 sun illumination in ambient environment. It is noted that after 32 h illumination, the control device only retained 61% of its initial efficiency. In comparison, the CL‐CH_3_ device maintained 75% of its initial efficiency after 40 h of measurement. While, for the CL‐CF_3_ device, over 80% of its initial efficiency was maintained after 72 h of measurement. The results suggest that CL‐CF_3_ complex can significantly enhance light stability of the device, which might be ascribed to the interaction between the complex and under‐coordinated Pb^2+^ ions, thus inhibiting ion migration under light illumination. **Figure** **5**a–c presents the long‐term, air and thermal stability of the unencapsulated devices, respectively. Clearly, OI complexes treated film‐based devices show superior stability over control device under the same condition. Specifically, after storage for 3000 h in an ambient environment (Figure 5b), the control, CL‐CH_3,_ and CL‐CF_3_ devices retained 73.2%, 84.5%, and 91% of their initial efficiency, respectively. This significant improvement of stability can be ascribed to three factors. First, the OI complexes treatment can increases the hydrophobicity of the resulting perovskite films as indicated by the increment of contact angle from 59.5° for control film to 73.3° for CL‐CH_3_ film then to 93.3° for CL‐CF_3_ film, as shown in Figure 5d–f, thus inhibiting permeation of moisture into the perovskite film.^[^ ^51^, ^52^, ^53^ ^]^ Second, the strong interaction between OI complexes and perovskite can suppress ion migration, thereby enhancing the thermal stability. Third, the dipole moment induced by the OI complexes can inhibit ion migration from HTL (such as Spiro‐OMeTAD) to perovskite, which is essential for the long‐term stability of the devices.

## 3 Conclusion

In summary, we have adopted a series of OI complexes as multifunctional interlayer materials for highly efficient and stable PSCs that fabricated by two‐step sequential method. Theoretical and experimental results suggest that this interlayer not only passivates the trap states of the perovskite films via interaction with under‐coordinated metal ions but also introduces dipole moments that synchronized with the built‐in electric field of the devices. Besides, it was demonstrated that a vertical dipole moment that is parallel with the built‐in electric field reduces the work function of perovskite film, modifies the interface energy level, and enhances the charge carrier extraction efficiency. As a result, through rational design of OI complex as an interfacial layer, devices with a champion PCE as high as 23.55% were fabricated. More importantly, the hydrophobicity of OI complexes remarkably improves the stability of the resulting devices.

## Conflict of Interest

The authors declare no conflict of interest.

## Supporting information

Supporting Information

## Data Availability

The data that support the findings of this study are available from the corresponding author upon reasonable request.
