# Supplementary material for: Anchoring Vertical Dipole to Enable Efficient Charge Extraction for High‐Performance Perovskite Solar Cells
Source: Adv Sci (Weinh). 2022 Sep 4;9(29):2203640. doi: 10.1002/advs.202203640 (PMC9561812; doi:10.1002/advs.202203640)
Supplement: Supplementary file 1 — Supporting Information [file ADVS-9-2203640-s001.pdf]

Supporting Information

Anchoring Vertical Dipole to Enable Efficient Charge Extraction for High Performance Perovskite Solar Cells

Heng Liu, Zhengyu Lu, Weihai Zhang, Jiantao Wang, Zhengli Lu, Quan Dai, Xingnan Qi, Yueqing Shi, Yuhui Hua, Rui Chen, Tingting Shi, Haiping Xia* and Hsing-Lin Wang**

Heng Liu
School of Materials Science and Engineering,
Harbin Institute of Technology, Harbin 150001, China

Heng Liu, Dr. Weihai Zhang, Dr. Jiantao Wang, Xingnan Qi, Prof. Hsing-Lin Wang*
Department of Materials Science and Engineering,
Southern University of Science and Technology,
Shenzhen 518055, Guangdong, China
E-mail:wangxl3@sustech.edu.cn

Hsing-Lin Wang*
Key University Laboratory of Highly Efficient Utilization of Solar Energy and Sustainable Development of Guangdong,
Southern University of Science and Technology,
Shenzhen 518055, Guangdong, China

Zhengyu Lu, Quan Dai, Yuhui Hua, Prof. Haiping Xia*
Shenzhen Grubbs Institute and Department of Chemistry,
Southern University of Science and Technology,
Shenzhen 518055, Guangdong, China
E-mail: xiahp@sustech.edu.cn

Zhengli Lu, Tingting Shi*
Guangdong Provincial Engineering Technology Research Center of Vacuum Coating Technologies and New Energy Materials,
Department of Physics,
Jinan University,
Guangzhou, Guangdong 510632, China
E-mail: ttshi@email.jnu.edu.cn

Yueqing Shi, Prof. Rui Chen,
Department of Electrical and Electronic Engineering,
Southern University of Science and Technology,

Shenzhen 518055, Guangdong, China

Experimental Section

Materials: ITO glass were purchased from Advanced Election Technology (China). SnO_2 were purchased from Alfa Aesar. Spiro-OMeTAD, Formamidinium Iodide (FAI), Methylammonium Bromide (MABr), Methylammonium Chloride (MACl) and Lithium-bis (trifluoromethanesulfonyl) imide (Li-TFSI) were purchased from Xi'an Polymer Light Technology Corp (Xi'an p-OLED). Lead (II) iodide PbI_2 were purchased from Advanced Election Technology (China). N,N-dimethylformamide (DMF), dimethylsulfoxide (DMSO), chlorobenzene (CB), isopropyl alcohol (IPA) ,acetonitrile (ACN) and Ethanol purchased from from Sigma-Aldrich. Gold (Au, 99.99%) were obtained from commercial sources.

Solution Preparation: SnO_2 colloid solution (15 wt %) was diluted within DI water (1:5.5, v: v), and stirring at room temperature for 10min, followed use a syringe and an aqueous filter to filter the above solution. The preparation of PbI_2 precursor solution, 599.3 mg PbI_2 powder was dissolved in 1mL DMF/DMSO 950:50 and stirred overnight at 70 ℃. The preparation of the solution of organic amine salts ， an isopropyl alcohol (IPA) solution containing organic salts (the mass ratio of FAI: MABr: MACl is 60 mg:6 mg: 6 mg), and stirred at 70 ℃for 30 min. The preparation of the solution of Spiro-OMeTAD HTL, which consisted of 72.3 mg spiro-OMeTAD, 28.8 µL 4-tertbutylpyridine, 17.5 µL lithium-bis (trifluoromethanesulfonyl) imide (Li-TFSI) solution (520 mg Li-TFSI in 1 mL acetonitrile), and 1 mL chlorobenzene. The preparation of organic-inorganic (OI) complexes solution, 1mg CL-CH_3, CL-CF_3 powder was dissolved in 1mL ethanol and stirred at room temperature.

Device Fabrication: The glass/ITO substrate was first scrubbed in detergent, and then sequentially cleaned by sonication in deionized water, acetone, and isopropanol for 25 minutes, respectively. After that, nitrogen dried glass/ITO were treated with plasma for 5 min before usage. Then deposit SnO_2 on the substrate as an electron transport layer by spin-coated at 3500 rpm for 30 s and annealed in ambient air at 150 ℃ for 30 min on the hot plate. After cooling to room temperature, the substrates were transferred to a nitrogen filled glove box. Then, 1.3M of PbI_2 dissolved in anhydrous DMF: DMSO 95:5(v:v) was spin coated onto SnO_2 at 2000 rpm for 30 s, and heating at 70 ℃ for 1 min and cooling 10 min, followed the mixture solution of FAI: MABr: MACl (60 mg:6 mg :6 mg in 1 mL IPA) was spin-coating onto the PbI_2 at 3000 rpm for 30 s, then transferred into dry air (10-20 RH%) filled glove box annealed at 150 ℃ for 10 min on the hot plate. After the substrates were transferred to a nitrogen filled glove box, cooling to room temperature. The dissolved CL-CH_3 and CL-CF_3 are spin-coated on the surface of the perovskite at 4000 rpm for 30 s. Subsequently, the substrate transferred into nitrogen filled glove box was deposited on the perovskite films as the hole transport layer by spin-casting the Spiro-OMeTAD solution at 4000 rpm for 30 s. Finally, an approximate 80 nm thick of Au electrode was fabricated using a shadow mask under high vacuum by thermal evaporation.

Density functional theory Methodology: Density functional theory (DFT) calculations were performed utilizing the Vienna Ab-initio Simulation Package (VASP) code with the projector augmented wave method (PAW). The generalized gradient approximation (GGA) of Perdew-Burke-Ernzerhof (PBE) was chosen for electron exchange and correlation. Energy cutoff, energy and force convergence criteria

were set at 450eV， 10^{-5}eV and 0.02eV $Å^{-1}$ respectively in all calculations. We used a 2×2×1 gamma centered k-point grid generated by the Monkhorst-Pack scheme for two passivation models of $FAPbI_3$ (001) surface structures (368 atoms) passivated by CL-CH_3 and CL-CF_3 molecules. All electrostatic potential (ESP) of molecules was calculated based on all-electron double-ξ valence basis sets of LanL2DZ.

Characterizations: The crystal structure and phase of the perovskite were characterized using X-ray diffraction spectrometer were obtained on Bruker Advanced D8 X-ray diffractometer using Cu Kα (λ = 0.154 nm) radiation. A UV-Vis spectrophotometer (Agilent Cary 5000) was used to collect the absorbance spectra of the perovskite films. Steady state photoluminescence (PL) spectra were recorded on Shimadzu RF-5301pc. Time-resolved photoluminescence spectra were measured on a PL system (Fluo-Time 300) under excitation with a picosecond pulsed diode laser with a repetition frequency of 1 MHz. The morphology of the films was studied by field-emission scanning electron microscopy (SEM; TESCAM MIRA3). The surface potential of perovskite films obtained with a atomic force microscope (AFM; Asylum Research MFP-3D-Stand Alone). An FEI Helios Nanolab 600i dual beam, focus ion beam/field emission gun-scanning electron microscope (FIB/FEGSEM) (FEI, Netherland), was used to prepare the device cross-section for scanning transmission electronic microscopy (STEM) imaging and analysis (FEI, Netherland). X-ray photoelectron spectroscopy (XPS) was conducted on a Thermo ScientificTM K-AlphaTM+ spectrometer equipped with a monochromatic Al Kα X-ray source (1486.6 eV) operating at 100 W. Samples were analyzed under vacuum (P < 10−8 mbar) with a pass energy of 150 eV (survey scans) or 50eV (high-resolution scans). The XPS spectra were calibrated by the binding energy of 284.8 eV for C 1s. Ultraviolet photoelectron spectroscopy (UPS, ESCALAB 250Xi, Thermo Fisher) measurements were carried out using a He Iα photon source (21.22 eV). The current density–voltage (*J*–*V*) curves of fabricated devices were obtained from the forward and reverse scan with 10 mV intervals and 10 ms delay time under AM 1.5 G illumination (100 mW cm^{-2}) were collected using a source meter (Keysight B2901A) and a solar simulator (Enlitech SS-F5-3A). The EQE spectra was measured using an quantum efficiency measurement system (Enlitech QER-3011) in which the light intensity at every wavelength was calibrated with a Si detector before measurement. The maximum-power point (MPP) output was measured by testing the steady-state current density at the maximum-power-point voltage. Electrochemical impedance spectroscopy (EIs) was tested with the frequency range from 100 Hz to 1 MHz by the electrochemical workstation (Princeton Applied Research, P4000+) in the dark conditions at with a bias of 1 V. The amplitude is 10 mV.

Statistical Analysis: In order to facilitate comparison, part of the data is normalized, such as the time-resolved PL (TRPL) results (Figure 4b in the revised manuscript) and the stability results (Figure 5a-c and Figure S24 in the revised manuscript). The statistical results were obtained by presenting the photovoltaic parameters of different number of devices. The sample size, specific test, and data presentation of each statistical analysis was specified in the corresponding figure legend. The TRPLcurves were fitted using OriginLab software. The scale bars of SEM, TEM and AFM images (Figure S17, Figure S18 and Figure S19 in the revised manuscript) were presented in the corresponding figure and specified in the figure legend. Specific details for all methods and softwares are discussed in the Characterization Section. Relavent content has been added in the revised manscript on page .

Preparation and characterization of organic-inorganic (OI) complexes

General information

All syntheses were carried out under an inert atmosphere (nitrogen or argon) using standard Schlenk techniques unless otherwise stated. Solvents were distilled under nitrogen from sodium/benzophenone (diethyl ether) or calcium hydride (dichloromethane) prior to use. The osmapentalyne **1** was synthesized according to the published literatures[1]. The other reagents and solvents were used as purchased from commercial sources without further purification. Column chromatography was performed on silica gel (200–300 mesh) in air. NMR spectra was collected on a Brucker AVANCE NEO 400 spectrometer (400 MHz) or Brucker AVANCE NEO 600 spectrometer (600 MHz). ^{1}H and ^{13}C{^{1}H} NMR chemical shifts (δ) are relative to tetramethylsilane, and ^{31}P{^{1}H} NMR chemical shifts are relative to 85% H_3PO_4. Two-dimensional and one-dimensional NMR spectra are abbreviated as HSQC (heteronuclear single quantum coherence), HMBC (heteronuclear multiple bond coherence) and DEPT (distortionless enhancement by polarization transfer). The absolute values of the coupling constants are given in hertz (Hz). Multiplicities are abbreviated as s (singlet), d (doublet), t (triplet), q (quartet), m (multiplet) and br (broad). The high-resolution mass spectra (HRMS) experiments were performed on a Thermo Scientific Q Exactive instrument.

Preparation and characterization of CL-CH_3

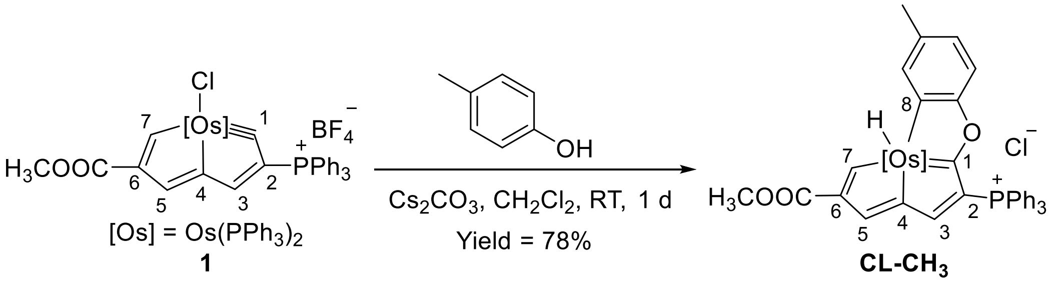

A mixture of compound **1** (120 mg, 0.10 mmol), 4-methylphenol (54 mg, 0.50 mmol) and Cs_2CO_3 (163 mg, 0.50 mmol) in 10 mL dichloromethane was stirred at RT for 1 d to give a red solution, and then the solid suspension was removed by filtration. The volume of the filtrate was reduced under vacuum to approximately 2 mL, and then loaded on silica gel column eluted by dichloromethane/methanol (20/1). The red band was collected, and the solvent was evaporated to dryness under vacuum to give a red solid. Yield, 99 mg, 78%.

^{1}H NMR plus ^{1}H-^{13}C HSQC (600.1 MHz, CD_2Cl_2): δ = 12.51 (d, J_{HH} = 13.3 Hz 1H, H7), 8.88 (m, 1H, H3), 8.84 (s, 1H, H5), 3.51 (s, 3H, COOC*H*$_3$), 1.85 (s, 3H, PhC*H*$_3$), -3.01 (td, J_{PH} = 17.2 Hz, J_{HH} = 13.2 Hz,1H, Os*H*), 7.90–6.25 ppm (48H, other aromatic protons). ^{31}P NMR (242.9 MHz, CD_2Cl_2): δ = 9.01 (s, C*P*Ph$_3$), -0.25 ppm (s, Os*P*Ph$_3$). ^{13}C NMR plus DEPT-135, ^{1}H-^{13}C HSQC and ^{1}H-^{13}C HMBC (150.9 MHz, CD_2Cl_2): δ = 251.4 (m, C1), 202.7 (t, J_{PC} = 12.5 Hz, C7), 194.9 (d, J_{PC} = 27.2 Hz, C4), 167.8 (d, J_{PC} = 15.5 Hz, C3), 163.7 (s, *C*OOCH$_3$), 157.9 (s, C5), 157.1 (s, C6), 136.5 (t, J_{PC} = 9.0 Hz, C8), 123.0 (d, J_{PC} = 73.8 Hz, C2), 50.8 (s, COO*C*H$_3$), 20.3 (s, Ph*C*H$_3$), 166.9–111.1 ppm (other aromatic carbons). HRMS (ESI): *m/z* calcd for $[C_{70}H_{58}O_3OsP_3]^+$, 1231.3208; found, 1231.3209.

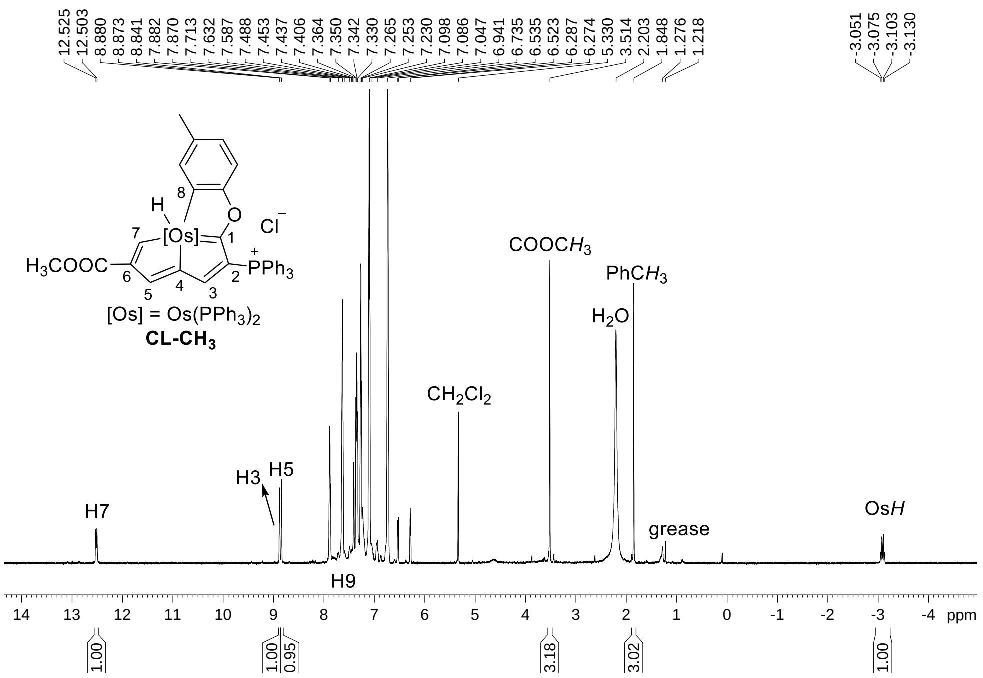

Figure S1. The ^{1}H NMR (600.1 MHz, CD_2Cl_2) spectrum of complex **CL-CH$_3$**.

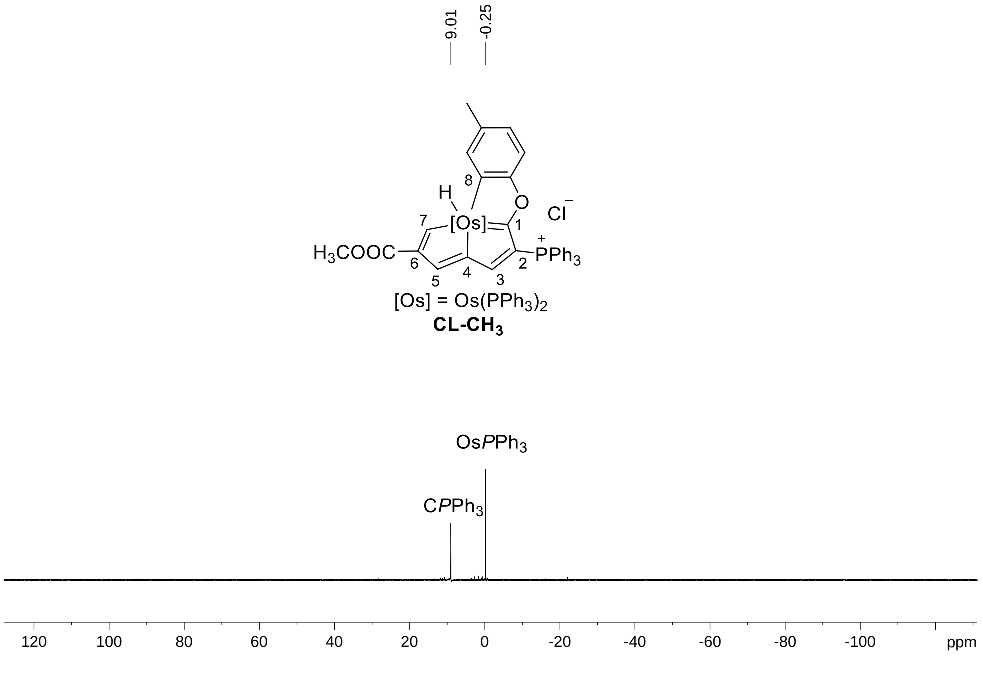

Figure S2. The $^{31}P\{^{1}H\}$ NMR (242.9 MHz, CD_2Cl_2) spectrum of complex **CL-CH$_3$**.

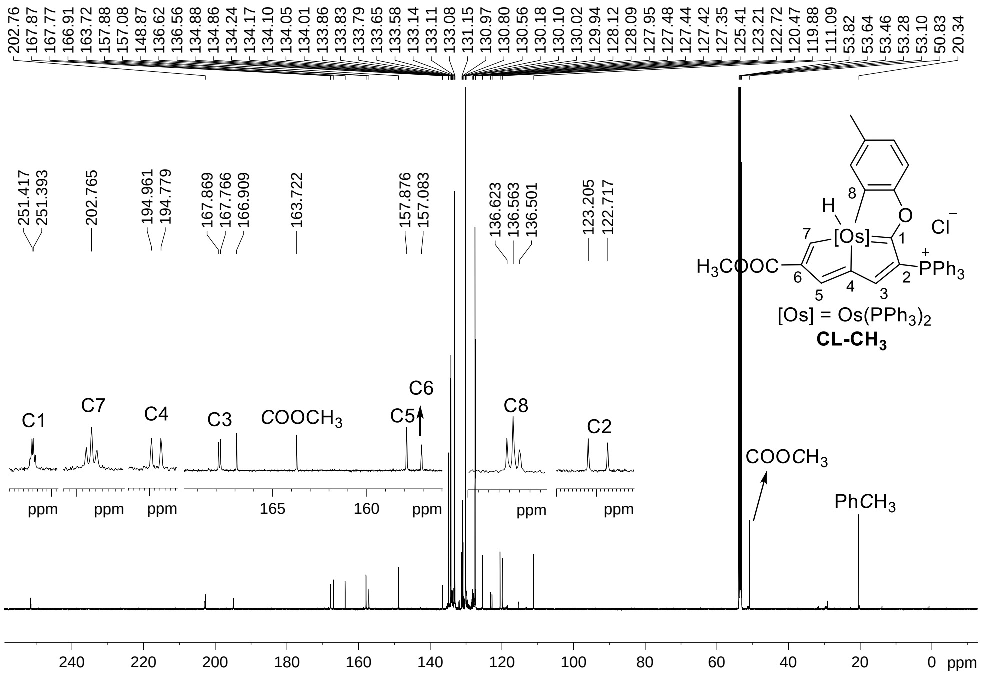

Figure S3. The $^{13}C\{^{1}H\}$ NMR (150.9 MHz, CD_2Cl_2) spectrum of complex **CL-CH$_3$.**

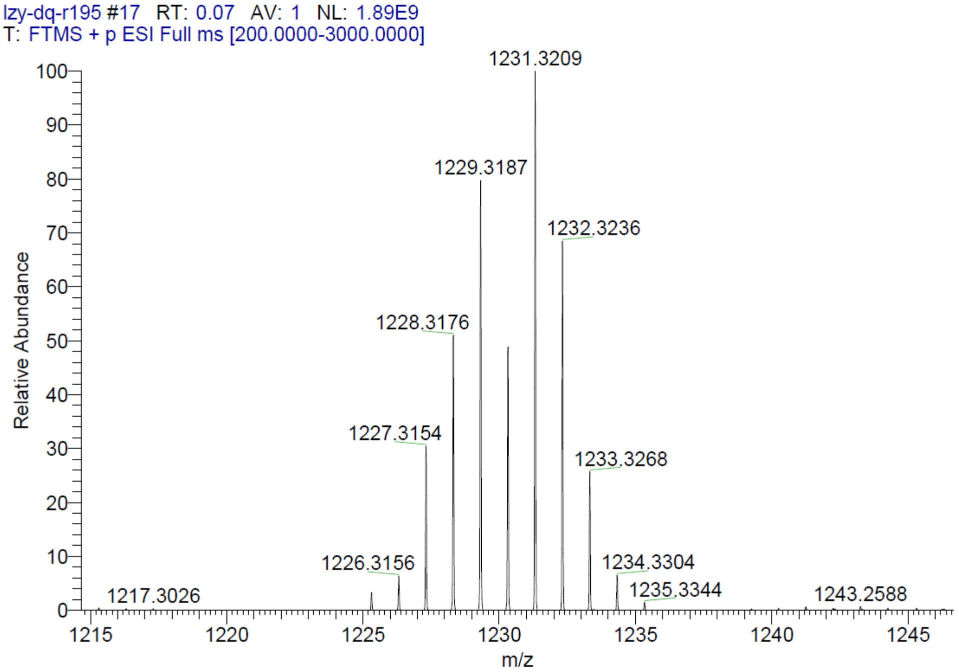

Figure S4. Positive-ion ESI-MS spectrum of [**CL-CH$_3$**]$^+$ measured in methanol.

Preparation and characterization of CL-CF$_3$

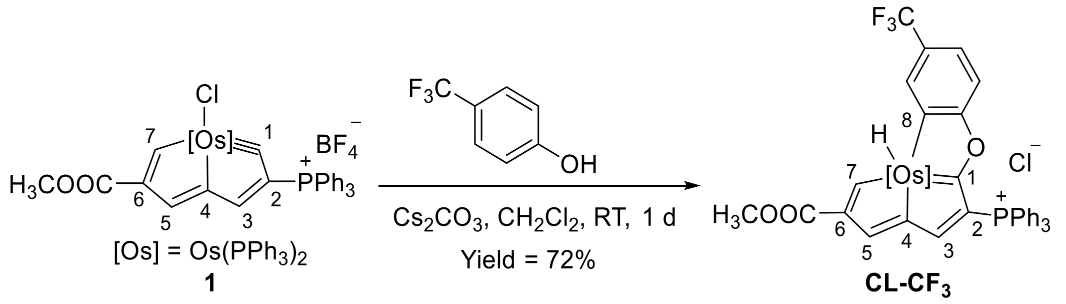

A mixture of compound **1** (120 mg, 0.10 mmol), 4-trifluoromethylphenol (81 mg, 0.50 mmol) and Cs_2CO_3 (163 mg, 0.50 mmol) in 10 mL dichloromethane was stirred at RT for 1 d to give a red solution, and then the solid suspension was removed by filtration. The volume of the filtrate was reduced under vacuum to approximately 2 mL, and then loaded on silica gel column eluted by dichloromethane/methanol (20/1). The red band was collected, and the solvent was evaporated to dryness under vacuum to give a red solid. Yield, 95 mg, 72%.

^{1}H NMR plus ^{1}H-^{13}C HSQC (600.1 MHz, CD_2Cl_2): δ = 12.70 (d, J_{HH} = 13.3 Hz 1H, H7), 9.05 (m, 1H, H5), 8.98 (d, J_{PH} = 4.7 Hz, 1H, H3), 3.55 (s, 3H, COOC*H*$_3$), -2.95 (td, J_{PH} = 16.7 Hz, J_{HH} = 13.5 Hz,1H, Os*H*), 7.96–6.32 ppm (48H, other aromatic protons). ^{31}P NMR (242.9 MHz, CD_2Cl_2): δ = 9.05 (s, C*P*Ph$_3$), 0.13 ppm (s, Os*P*Ph$_3$). ^{19}F NMR (564.7 MHz, CD_2Cl_2): δ = -61.84 ppm (s, C*F*$_3$).^{13}C NMR plus DEPT-135, ^{1}H-^{13}C HSQC and ^{1}H-^{13}C HMBC (150.9 MHz, CD_2Cl_2): δ = 251.0 (m, C1), 204.3 (t, J_{PC} = 12.6 Hz, C7), 194.9 (d, J_{PC} = 27.8 Hz, C4), 167.9 (d, J_{PC} = 15.5 Hz, C3), 163.6 (s, *C*OOCH$_3$), 159.4 (s, C5), 157.9 (s, C6), 138.7 (t, J_{PC} = 9.0 Hz, C8), 123.5 (d, J_{PC} = 73.6 Hz, C2), 51.0 (s, COO*C*H$_3$), 170.3–110.9 ppm (other aromatic carbons). HRMS (ESI): *m/z* calcd for $[C_{70}H_{55}F_3O_3OsP_3]^+$, 1285.2925; found, 1285.2921.

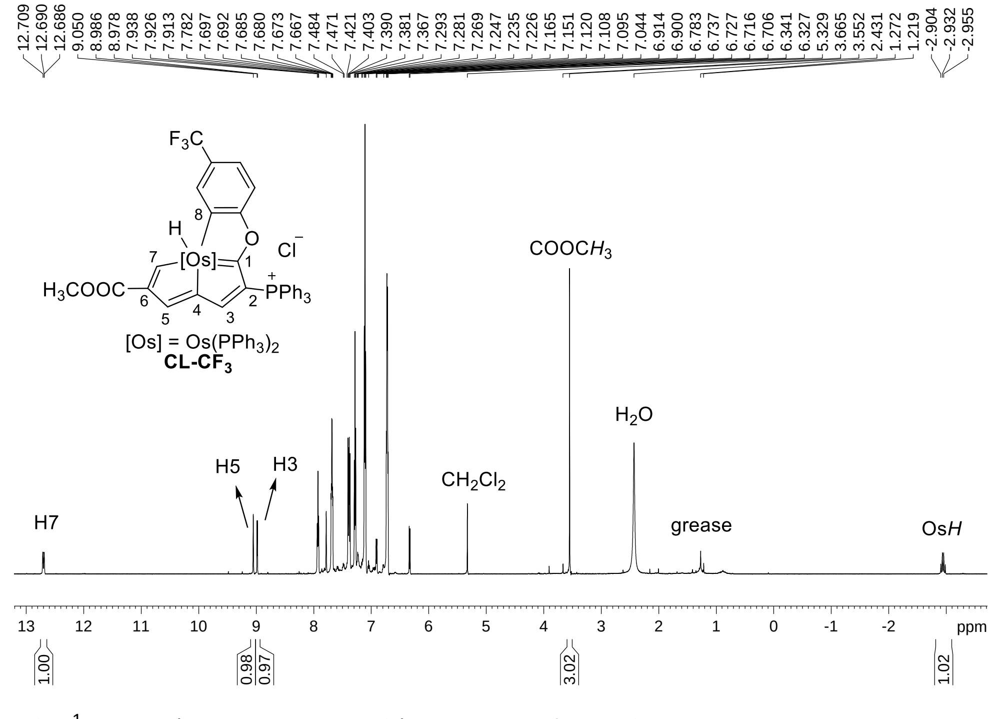

Figure S5. The ^{1}H NMR (600.1 MHz, CD_2Cl_2) spectrum of complex **CL-CF$_3$**.

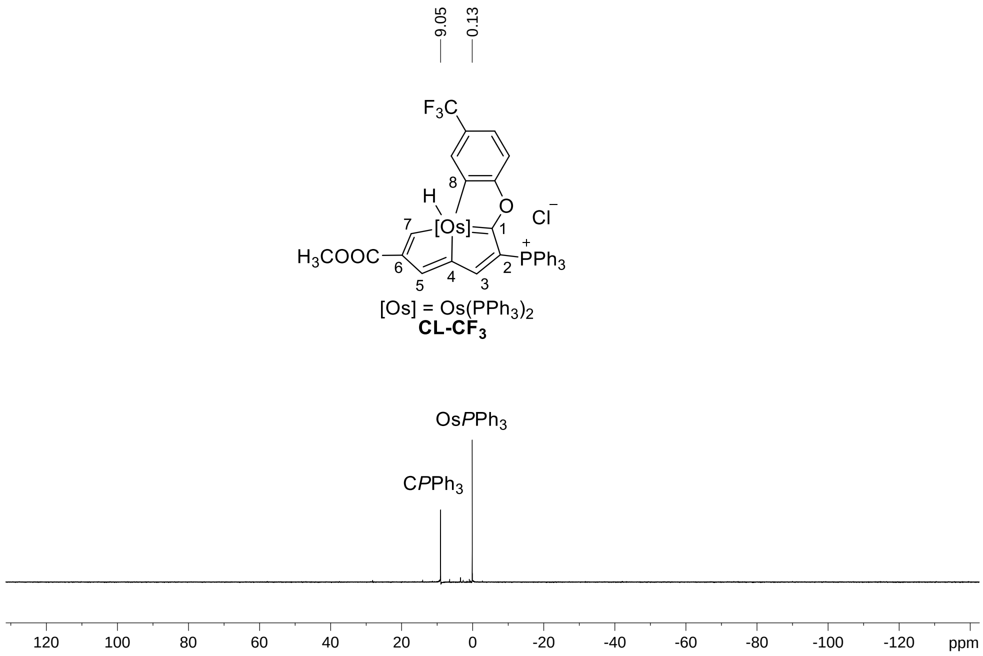

Figure S6. The $^{31}P\{^{1}H\}$ NMR (242.9 MHz, CD_2Cl_2) spectrum of complex **CL-CF$_3$**.

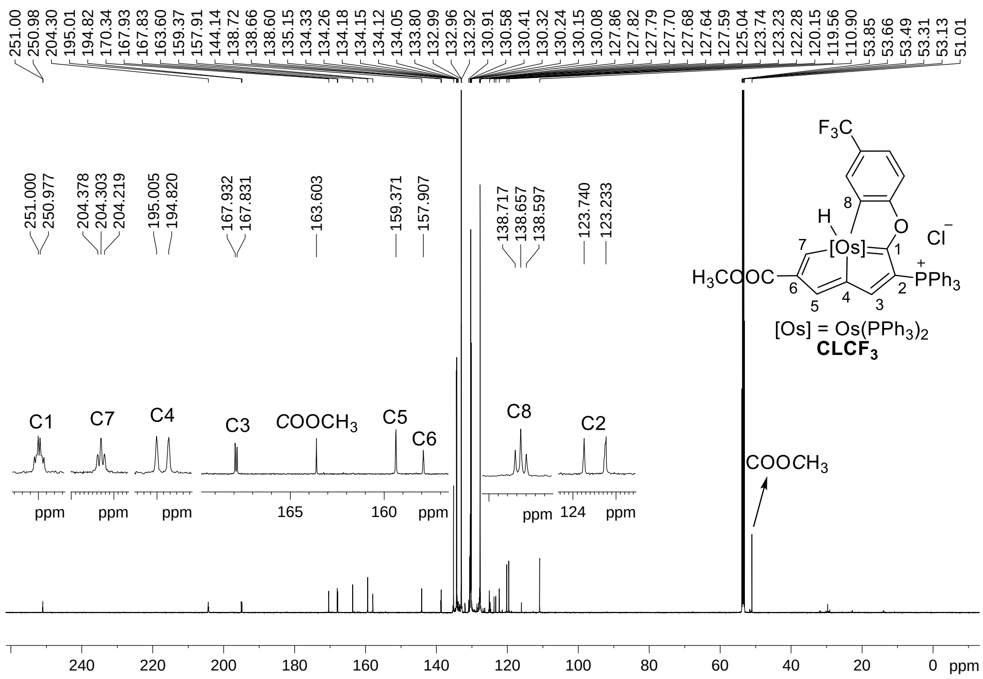

Figure S7. The $^{13}C\{^{1}H\}$ NMR (150.9 MHz, CD_2Cl_2) spectrum of complex **CL-CF$_3$**.

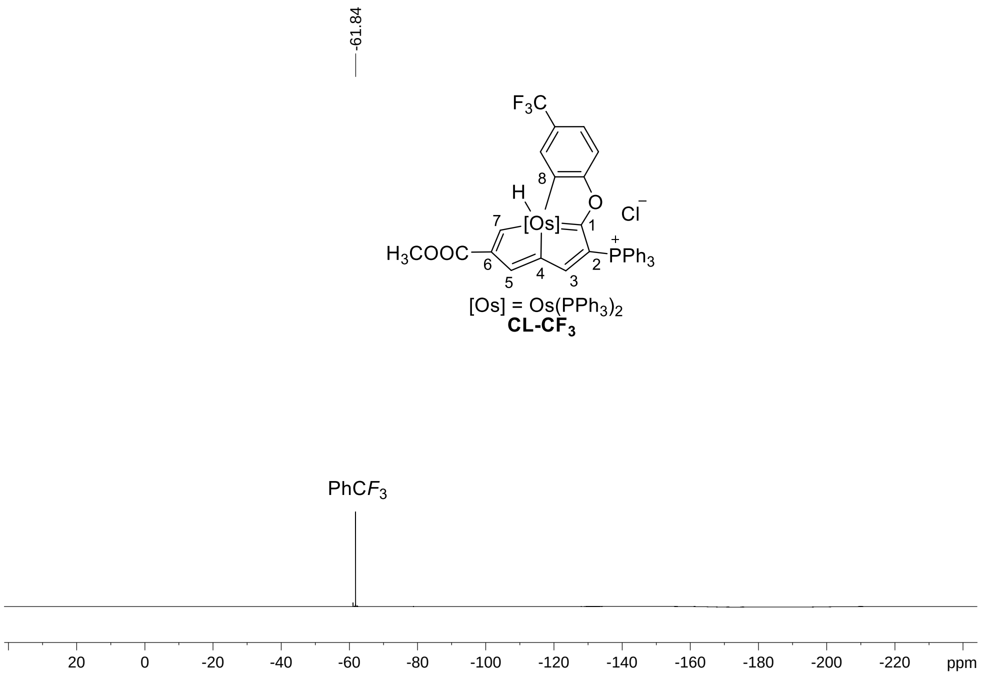

Figure S8. The $^{19}F\{^{1}H\}$ NMR (564.7 MHz, CD_2Cl_2) spectrum of complex **CL-CF$_3$**.

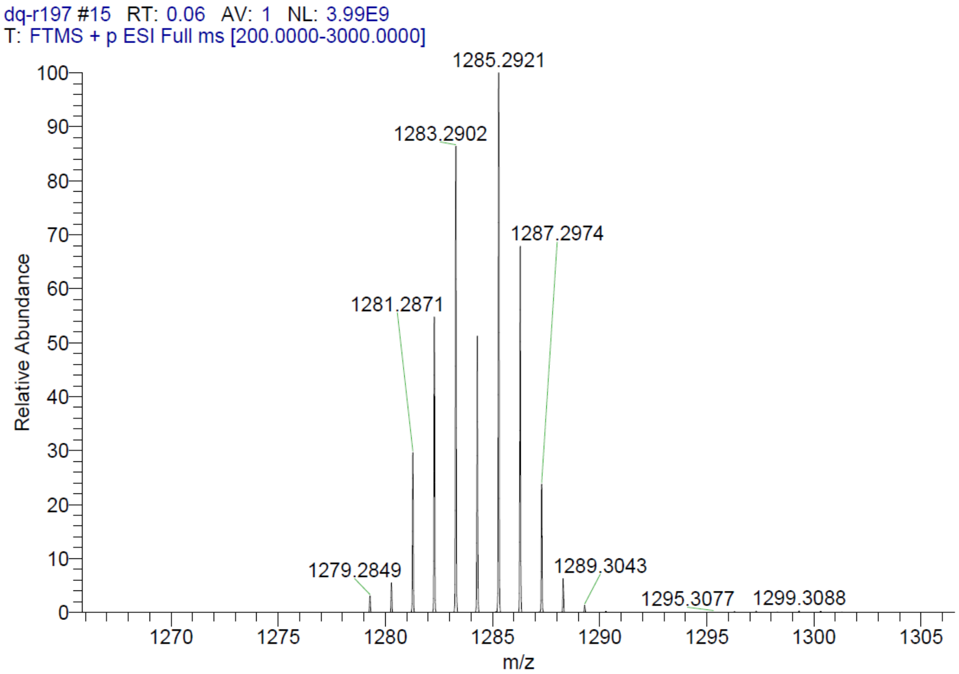

Figure S9. Positive-ion ESI-MS spectrum of [**CL-CF$_3$**]$^+$ measured in methanol.

X-ray Crystallographic Analysis

The single crystals suitable for X-ray diffraction was grown from chloroform solution layered with

hexane. Single-Crystal X-ray diffraction data were collected on a Bruker SMART APEX2 area detector diffractometer with a Cu Kα radiation (λ = 1.54184 Å). Multi-scan absorption corrections was applied for **CL-CH$_3$**. Using Olex2[2], the structure of **CL-CF$_3$** was solved with ShelXT[3] structure solution program using Intrinsic Phasing and refined with the ShelXL[4] refinement package using Least Squares minimization. All non-hydrogen atoms were refined anisotropically unless otherwise stated. Hydrogen atoms were placed at idealized positions and assumed the riding model. The diffuse electron densities resulting from the residual solvent molecules in complex **CL-CF$_3$** was removed from the data set by using the Solvent Mask routine of Olex2. CCDC-2160622 (**CL-CF$_3$**) contain supplementary crystallographic data for this paper. These data can be obtained free of charge from The Cambridge Crystallographic Data Centre.

Crystal Data for CL-CF$_3$: $C_{70}H_{55}ClF_3O_3OsP_3$ [$C_{70}H_{55}F_3O_3OsP_3$]Cl (*Mr* = 1319.70 g/mol): triclinic, crystal dimension 0.20 × 0.20 × 0.10 mm, space group P-1 (no. 2), *a* = 10.8099(5) Å, *b* = 12.8692(7) Å, *c* = 24.9865(11) Å, *α* = 88.596(2)°, *β* = 81.128(2)°, *γ* = 68.039(2)°, *V* = 3183.1(3) Å^3, *Z* = 2, *T* = 100.0 K, μ(CuKα) = 5.316 mm^{-1}, *Dcalc* = 1.377 g/cm^3, 57079 reflections measured (7.166° ≤ 2Θ ≤ 137.564°), 11620 unique (R_{int} = 0.0873, R_{sigma} = 0.0630) which were used in all calculations. The final R_1 was 0.0523 (I > 2σ(I)) and wR_2 was 0.1483 (all data). GOF = 1.115. Residual electron density (e. Å^{-3}) max/min: 2.55/-1.71.

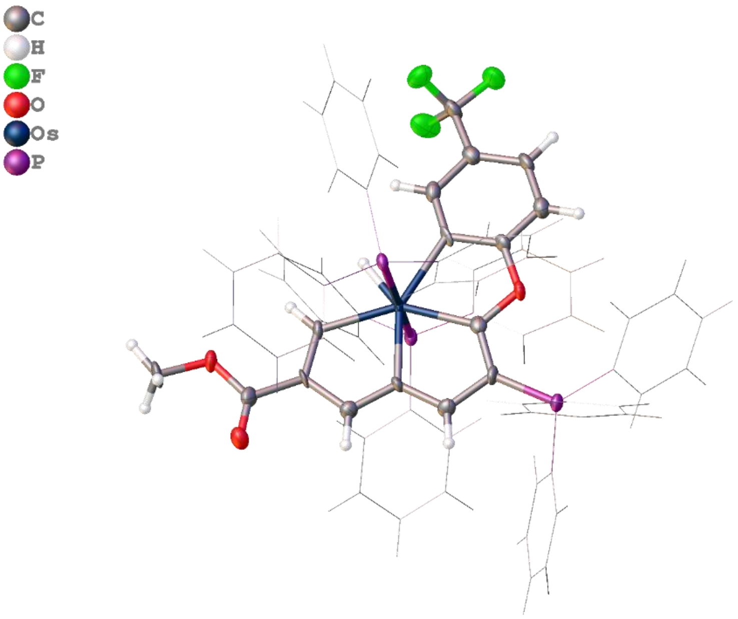

Figure S10. X-ray molecular structure for the cation of complex **CL-CF$_3$** drawn with 50% probability level.

Computational calculations

All the calculations were performed with the Gaussian 09 software package.[5] The M06L/6-31G* level with SDD for Os atom.[6,7] The energies of their molecular orbitals were further calculated at the B3PW91/def2-TZVP level.[8, 9]

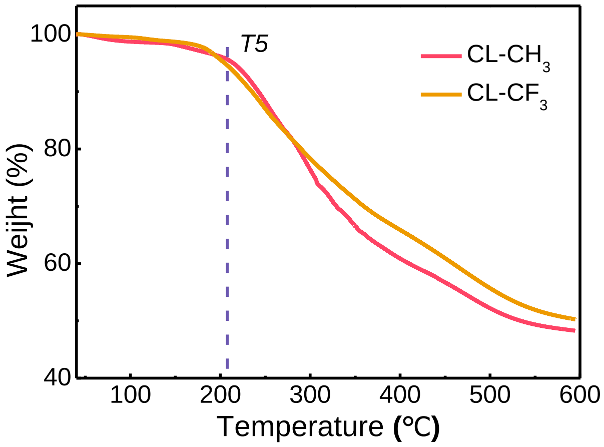

Figure S11. Thermogravimetric measurements of CL-CH_3 and CL-CF_3 complexes.

Note: Thermogravimetric analyses (TGA) of CL-CH_3 and CL-CF_3 show that the initial decomposition temperatures (T5) as measured at the point of 5% weight loss are 217.1 ℃ and 207.6 ℃, respectively.

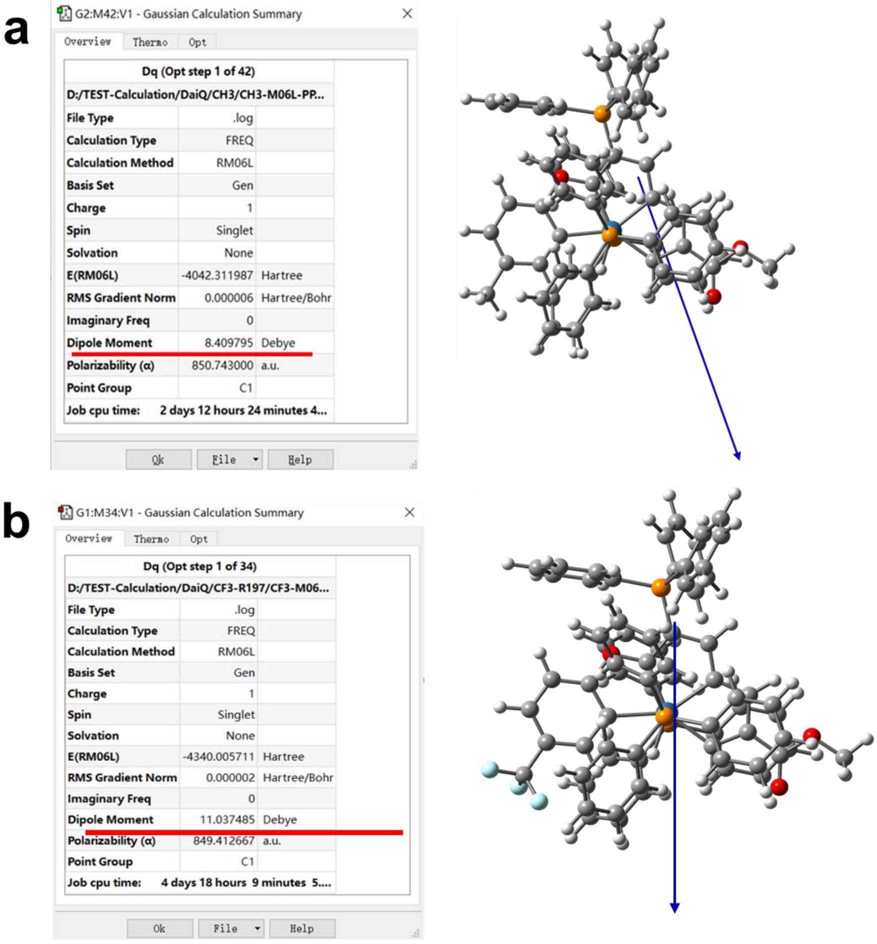

Figure S12. Gaussian calculation summary of CL-CH_3 and CL-CF_3 complex.

Note: According to density functional theory (DFT), the dipole moments of CL-CF3 and CL-CH3 are 8.4 D and 11.04 D, respectively. due to the existence of strong electron-withdrawing Trifluoromethyl (-CF_3) group, CL-CF_3 exhibits higher dipole moment of than that of CL-CH_3 due to the existence of strong electron-withdrawing Trifluoromethyl (-CF_3) group.

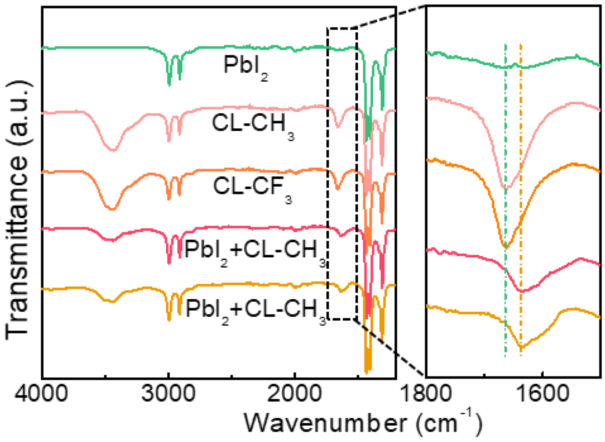

Figure S13. FTIR spectra and fingerprint regions of pure PbI_2, CL-CH_3, CL-CF_3, PbI_2 +CL-CH_3 and PbI_2 +CL-CF_3 mixture in DMSO.

Note: The stretching vibration of C=O bond shifted from 1660 cm^{-1} in pure CL-CH_3 and CL-CF_3 molecules to a lower wavenumber of 1643 cm^{-1} for the PbI_2+CL-CH_3 and PbI_2+CL-CF_3 samples.

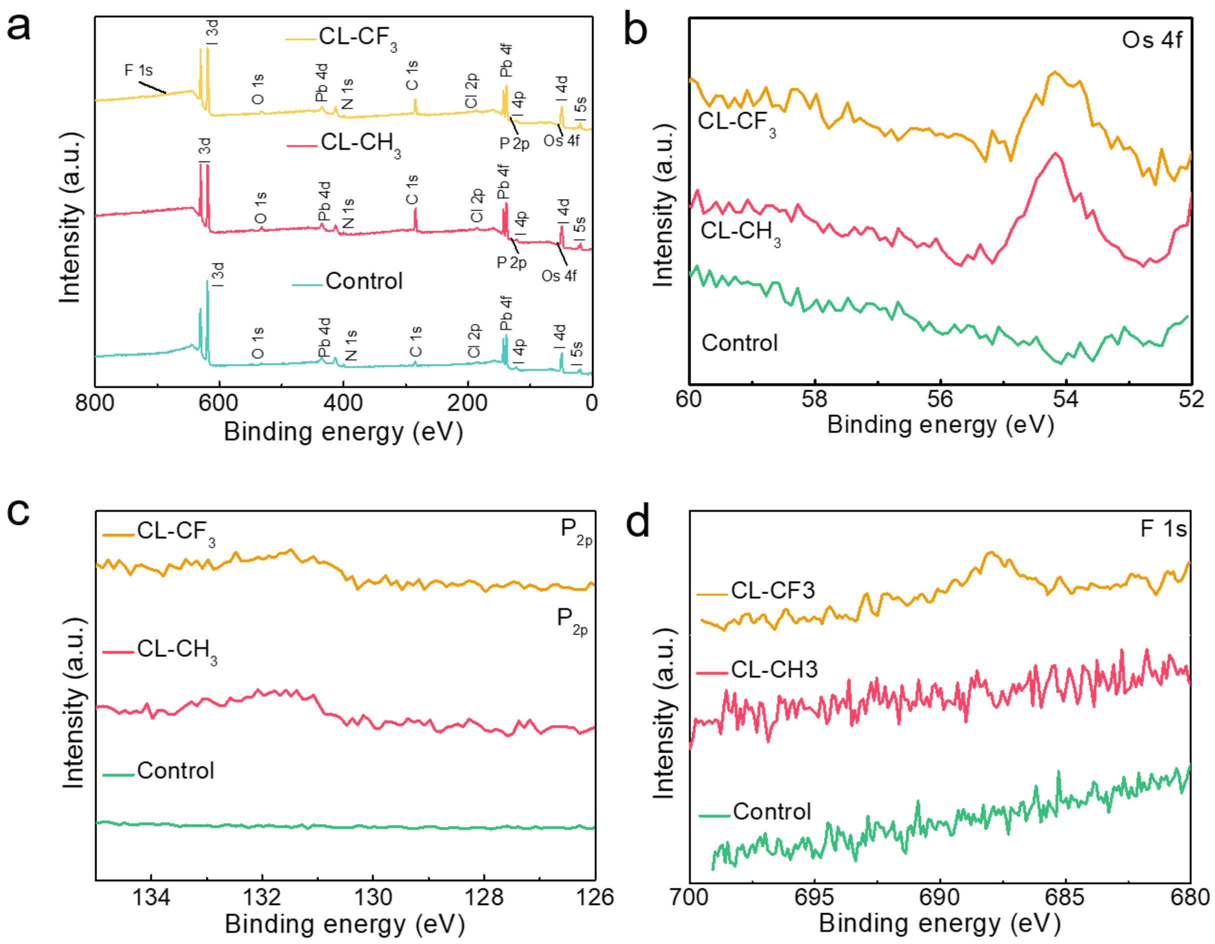

Figure S14. a) XPS survey spectra of control, CL-CH$_3$ and CL-CF$_3$ films. b-d) Os 4f core spectra, c) P 2p core spectra, and d) F 1s core spectra of control, CL-CH$_3$, CL-CF$_3$ films, respectively.

Note: It can be seen from the XPS survey spectra that control, CL-CH$_3$ and CL-CF$_3$ perovskite films all contain Pb, I, Cl, C, N, O. The appearance of Os 4f, P 2p and F 1s signal indicates the existence of OI complexes in the final film.

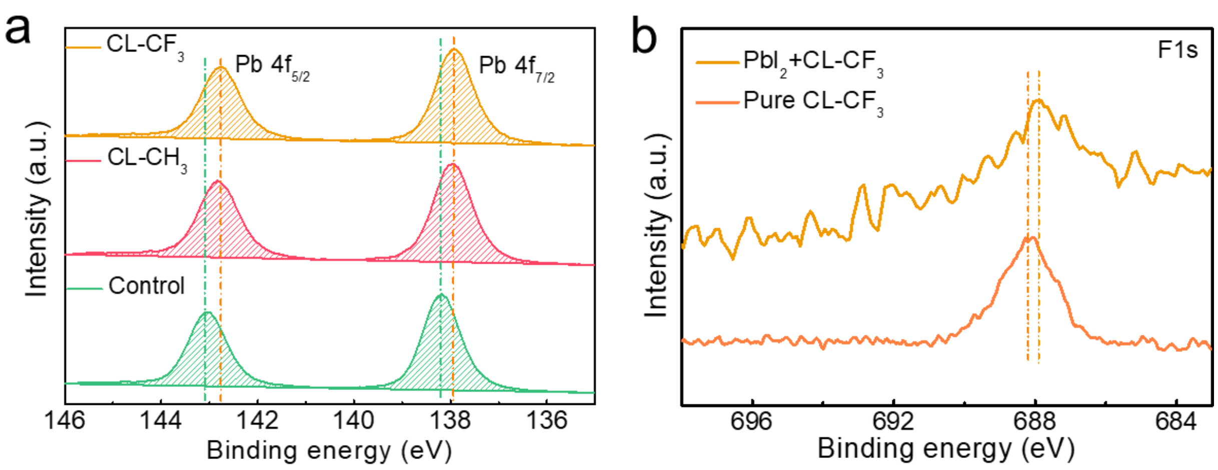

Figure S15. a) Pb 4f core spectra of perovskite films without and with CL-CH_3 and CL-CF_3 treatment. b) F 1s core spectra of PbI_2 +CL-CF_3 and CL-CF_3 film.

Note: The Pb 4f peaks of control film are consistent with the reported binding energies of constituent elements of perovskite material, where Pb $4f_{5/2}$ and Pb $4f_{7/2}$ peaks are 143.1 and 138.2 eV, respectively. In the case of Pb $4f_{5/2}$ and Pb $4f_{7/2}$ of CL-CH_3 and CL-CF_3 treatment perovskite film, the peaks are 142.8, 137.97 eV and 142.7, 137.94 eV, respectively. The F 1s peaks of pure CL-CF_3 are 688.2eV. The F 1s peaks of PbI_2+ CL-CF_3 are 687.9eV.

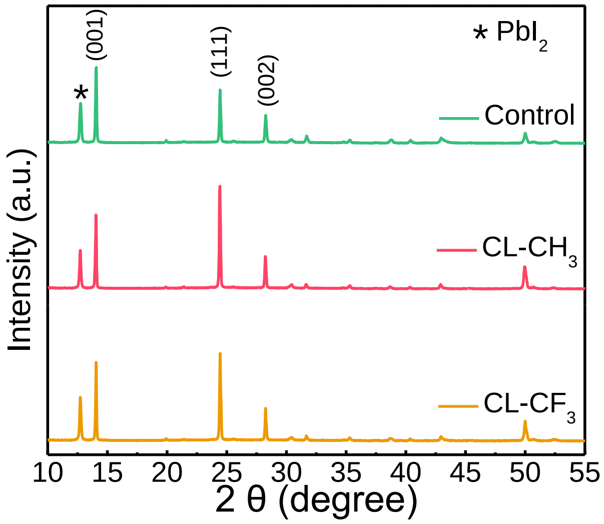

Figure S16. XRD patterns of CL-CH_3, CL-CF_3 modified and control perovskite films.

Note: The XRD results suggest that the OI complexes will not affect the crystallinization.

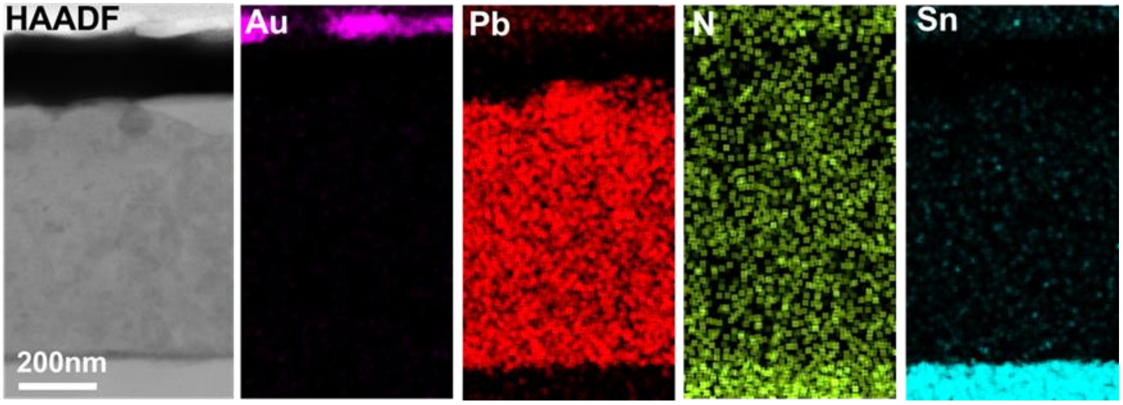

Figure S17. STEM mappings of PSCs device cross-sectional images.

Note: The cross-sectional scanning transmission electronic microscopy (STEM) image of the device was presented, where all the functional layers are clearly observed, the thickness of the perovskite film, which is ≈500 nm.

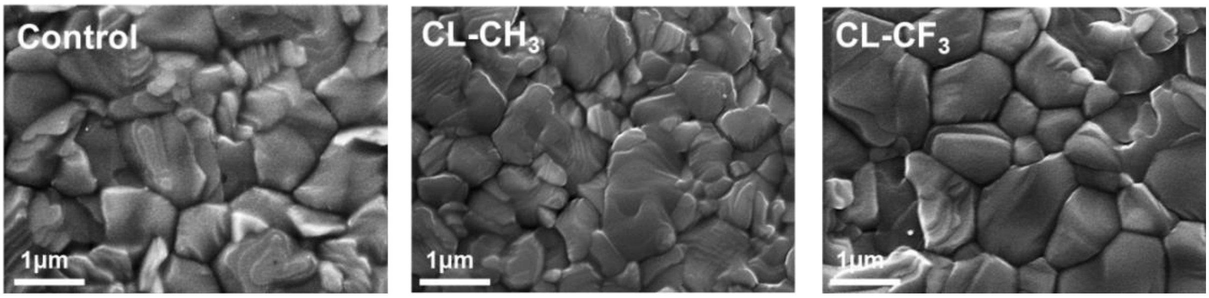

Figure S18. Surface SEM images of control, CL-CH_3, CL-CF_3 films.

Note: The SEM results suggest that the OI complexes will not affect the morphology.

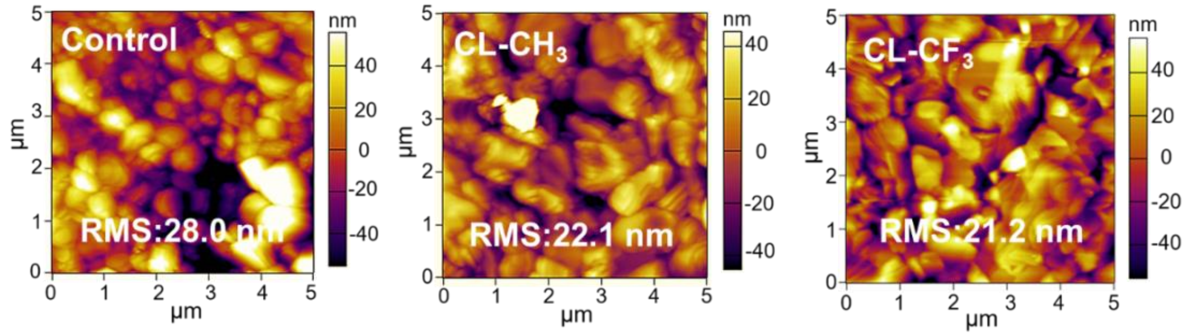

Figure S19. AFM image of control, CL-CH_3, CL-CF_3 films.

Note: The OI complexes treated films reveal smaller root mean square (RMS) values, which is helpful for charge extraction.

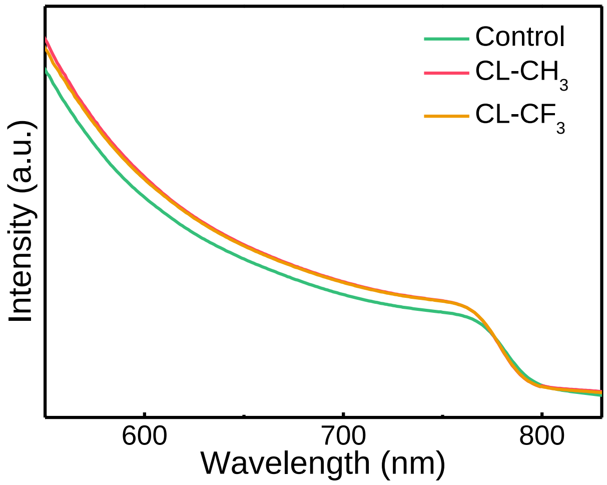

Figure S20. UV-Vis absorption spectra of control, CL-CH_3, CL-CF_3 films.

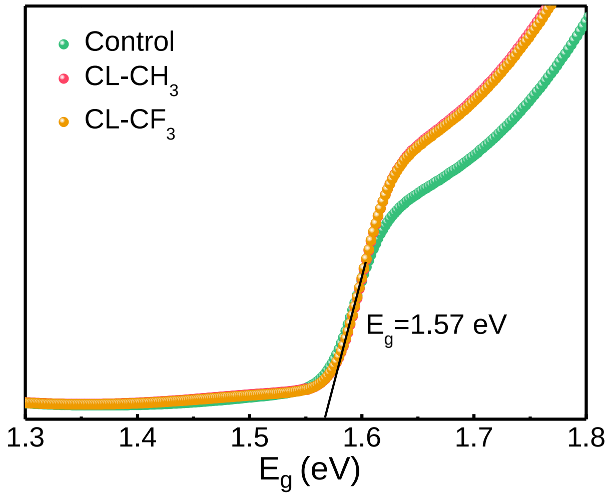

Figure S21. Tauc's plot calculated from the UV-Vis absorption spectra with equation (α h υ)^2= A(h υ - E_g), where α is the absorption coefficient, hυ is the photon energy, and E_g is the bandgap.

Note: The UV–vis spectrum implies that the presence of CL-CH_3, CL-CF_3 molecules does not affect the bandgap of the perovskite materials. However, the existence of CL-CH_3, CL-CF_3 molecules makes the UV-Vis absorption intensity enhanced.

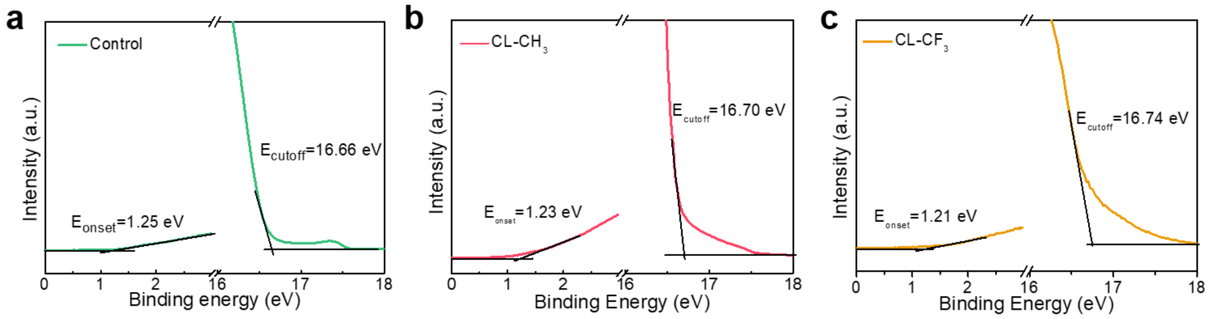

Figure S22. Ultraviolet photoelectron spectroscopy (UPS) of a) control, b) CL-CH_3 and c) CL-CF_3 films.

Note: Perovskite film the ionization energy (IE) according to equation IE= 21.22-[$E_{cut\text{-}off}$ - E_{onset}]. Combining with the optical bandgap derived from UV-vis absorption (Figure S19, Supporting Information), the position of conduction band minimum (CBM) and valence band maximum (VBM) of the films were ascertained, and the corresponding parameters are summarized in Table S1.

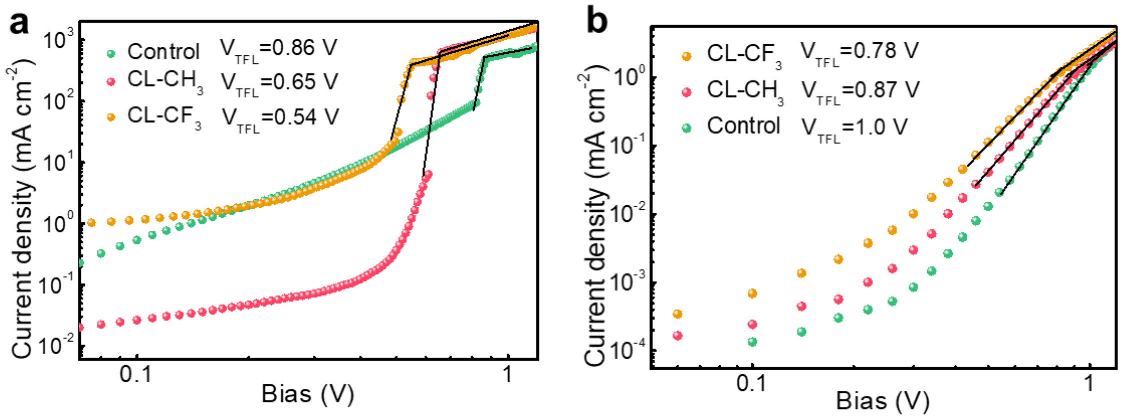

Figure S23. a) Dark J–V curves for the electron-only devices based on control, CL-CH_3 and CL-CF_3 films. b) Dark J–V curves for the hole-only devices based on control, CL-CH_3 and CL-CF_3 films.

Note: The typical dark *J-V* curves of electron-only devices with structure of ITO/SnO_2/Perovskite/PCBM/Ag. The typical dark J-V curves of hole-only devices with structure of ITO/NiOx/ Perovskite/Spir-OMeTAD/Au. According to $N_t = \frac{2V_{TFL}\varepsilon_r\varepsilon_0}{eL^2}$, where ε_r is the relative dielectric constant of perovskite, which is 62.23, ε_0 is the vacuum permittivity, e is the elementary charge, and L is the thickness of the perovskite film. It can be calculated that the electron-only devices trap-state density of control, CL-CH_3, and CL-CF_3 films are 2.37×10^{16}, 1.79×10^{16}, and 1.48×10^{16} cm^{-3}, respectively and the hole-only devices trap-state density of control, CL-CH_3, and CL-CF_3 films are 2.76×10^{16}, 2.41×10^{16}, and 2.14×10^{16} cm^{-3}, respectively. The electron mobility of the films was estimated using the Mott-Gurney equation as follows: $\mu = \frac{8J_DL^3}{9\varepsilon_0\varepsilon_rV^2}$, where JD and V are current density and voltage at the SCLC region. After carefully calculation, the hole mobility of control, CL-CH_3, and CL-CF_3 films located at 3.8×10^{-2}, 4.2×10^{-2}, and 5.7×10^{-2} $cm^{-2}V^{-1}S^{-1}$, respectively. The results suggest that CL-CF_3 complex can effectively improve the hole mobility of the film.

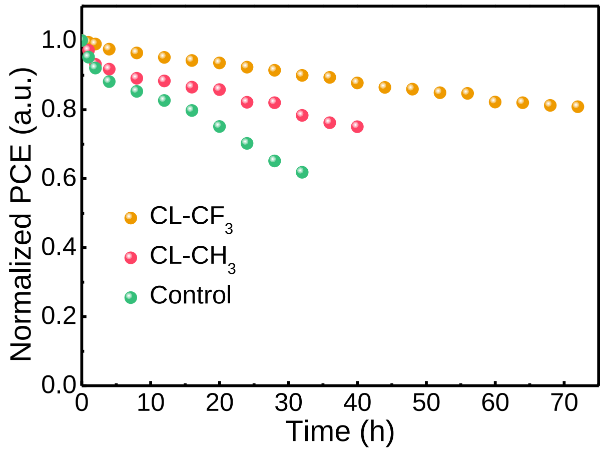

Figure S24 The operational stability of the corresponding unencapsulated devices under continuous 1 sun illumination in ambient environment.

Note: It is noted that after 32 hours illumination, the control device only retained 61% of its initial efficiency. In comparison, the CL-CH_3 device maintained 75% of its initial efficiency after 40 hours measurement. While, for the CL-CF_3 device, over 80% of its initial efficiency was maintained after 72 hours measurement.

Table S1. Conduction band minimum (CBM) and valence band maximum (VBM) of control, CL-CH_3 and CL-CF_3 films.

Sample	band gap [eV]	VBM [eV]	CBM [eV]
Control	1.57	5.81	4.24
CL-CH_3	1.57	5.75	4.18
CL-CF_3	1.57	5.69	4.12

Table S2. TRPL parameters of the control, CL-CH_3 and CL-CF_3 films.

Sample	A_1	τ_1 [ns]	A_2	τ_2 [ns]	τ_{ave} [ns]
Control	0.34	91.10	0.68	377.40	346.6
CL-CH_3	0.25	29.29	0.71	161.05	151.1
CL-CF_3	0.45	24.43	0.60	126.39	113.5

Note: The fitting biexponential decay function was $y = y_0 + A_1 exp\left(-\frac{t}{\tau_1}\right) + A_2 exp\left(-\frac{t}{\tau_2}\right)$, $\tau_{ave}= \frac{A_1 t_1^2 + A_2 t_2^2}{A_1 t_1 + A_2 t_2}$.

References

[1] Zhu, C.; Li, S.; Luo, M.; Zhou, X.; Niu, Y.; Lin, M.; Zhu, J.; Cao, Z.; Lu, X.; Wen, T.; Xie, Z.; Schleyer, P. v. R.; Xia, H. *Nat. Chem.* **2013**, *5*, 698-703.
[2] Dolomanov, O. V.; Bourhis, L. J.; Gildea, R. J.; Howard, J. A. K.; Puschmann, H. *J. Appl. Cryst.* **2009**, *42*, 339-341.
[3] Sheldrick, G. M. *Acta Cryst. Sect A* **2015**, *71*, 3-8.
[4] Sheldrick, G. M. *Acta Cryst. Sect C* **2015**, *71*, 3-8.
[5] *Gaussian 09, Revision D.01; Gaussian, Inc.: Wallingford, 2010.* Frisch, M. J.; Trucks, G. W.; Schlegel, H. B.; Scuseria, G. E.; Robb, M. A.; Cheeseman, J. R.; Scalmani, G.; Barone, V.; Mennucci, B.; Petersson, G. A.; Nakatsuji, H.; Caricato, M.; Li, X.; Hratchian, H. P.; Izmaylov, A. F.; Bloino, J.; Zheng, G.; Sonnenberg, J. L.; Hada, M.; Ehara, M.; Toyota, K.; Fukuda, R.; Hasegawa, J.; Ishida, M.; Nakajima, T.; Honda, Y.; Kitao, O.; Nakai, H.; Vreven, T.; Montgomery, J. A.; Peralta, Jr., J. E.; Ogliaro, F.; Bearpark, M.; Heyd, J. J.; Brothers, E.; Kudin, K. N. V. N.; Staroverov, Keith, T.; Kobayashi, R.; Normand, J.; Raghavachari, K.; Rendell, A.; Burant, J. C.; Iyengar, S. S.; Tomasi, J.; Cossi, Rega, N.; Millam, J. M.; Klene, M.; Knox, J. E.; Cross, J. B.; Bakken, V.; Adamo, C.; Jaramillo, J.; Gomperts, R.; Stratmann, R. E.; Yazyev, O.; Austin, A. J.; Cammi, R.; Pomelli, C.; Ochterski, J. W.; Martin, R. L.; Morokuma, K.; Zakrzewski, V. G.; Voth, G. A.; Salvador, P.; Dannenberg, J. J.; Dapprich, S.; Daniels, A. D.; Farkas, O.; Foresman, J. B.; Ortiz, J. V.; Cioslowski, J. and Fox, D. J. Gaussian, Inc., Wallingford CT, **2013**.
[6] Zhao, Y.; Truhlar, D. G. *J. Chem. Phys.* **2006**, *125*, 194101.
[7] Andrae, D.; Häußermann, U.; Dolg, M.; Stoll, H.; Preuß, H. *Theoret. Chim. Acta* **1990**, *77*, 123-141.

[8] Becke, A. D. *J. Chem. Phys.* **1993**, *98*, 5648-5652.
[9] Perdew, J. P.; Burke, K.; Wang, Y. *Phys. Rev. B* **1996**, *54*, 16533-16539.
